# CO_2_ and H_2_O Sorption Induced Bulk-Phase Changes of CALF-20 Captured Using *In Situ* Laboratory X‑ray Powder Diffraction

**DOI:** 10.1021/jacs.5c06866

**Published:** 2025-07-11

**Authors:** Sebastian Bette, Anastasia Sleptsova, Bettina V. Lotsch, Robert E. Dinnebier, Stefan Marx, Mahsa Loloei, Adebayo A. Adeleke, Nima Masoumifard, Ramanathan Vaidhyanathan

**Affiliations:** † 28326 Max Planck Institute for Solid State Research, Heisenbergstraße 1, 70569 Stuttgart, Germany; ‡ Department of Chemistry, Ludwig-Maximilians-Universität (LMU), Butenandtstrasse 5-13, 81377 Munich, Germany; § BASF SE, Carl-Bosch-Strasse 38, 67056 Ludwigshafen, Germany; ∥ Svante Inc., 8800 Glenlyon Pkwy, Burnaby, British Columbia V5J 5K3, Canada

## Abstract

The zinc oxalate (ox) triazolate (trz)-based MOF, Calgary Framework 20 (CALF-20), exhibits remarkable cycling stability for carbon dioxide and water adsorption and desorption and is therefore a promising candidate material for CO_2_ sequestration on an industrial scale. Upon gas and vapor loading and unloading, the MOF shows pronounced structural dynamics leading to a variety of potential CALF-20 polymorphs. A systematic *in situ* study on CO_2_ and H_2_O ad- and desorption using high-resolution, laboratory X-ray powder diffraction (XRPD) shows that the CO_2_-breathing behavior changes upon gas loading. A CO_2_ uptake initially distorts the rectangular pore into a diamond shape. Upon further CO_2_ incorporation, the breathing behavior changes, and the pore becomes more rectangular, again. At low temperatures (−70 °C), the uptake of CO_2_ occurs in a core–shell mechanism, and the gas is bound strongly to the framework and cannot be removed by dynamic vacuum. During water uptake of CALF-20, two distinct hydrated phases can be identified. The overall water loading capacity is independent of temperature between 25 and 60 °C. In this paper, we demonstrate that recent advances in X-ray powder diffraction hard- and software enable a detailed investigation of the loading and breathing behavior of a crystalline MOF using laboratory equipment, turning this into easily accessible investigations.

## Graphical abstract

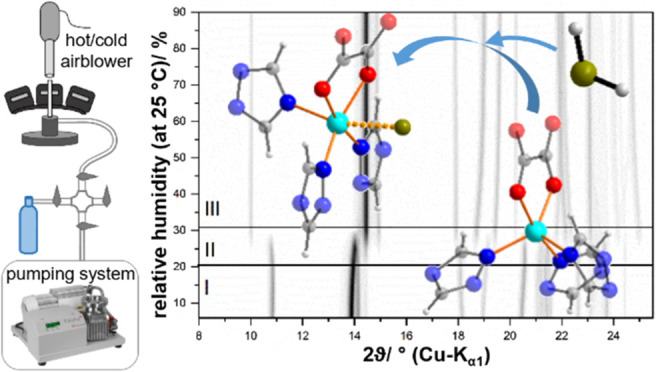

## 1 Introduction

In recent years, metal–organic frameworks (MOFs) have attracted broad interest in the scientific community due to their literally infinite design space in combining a large number of organic linkers with a variety of metal nodes. This structural flexibility opens up many possible applications for MOFs, ranging from heterogenic catalysis^2^ over adsorber materials for gaseous nerve agents^3^ to carriers for active pharmaceutical components.^4^ Among these promising applications, gas adsorption and storage, particularly in adsorbing CO_2_ from combustion and atmospheric gases, has the highest potential and relevance for large-scale realization.^5^ In 2021, Lin and co-workers^6^ reported a zinc oxalate (ox) triazolate (trz)-based MOF called Calgary Framework 20 (CALF-20), Zn_2_(ox)­(trz)_2_·*n* guest molecules, which shows a remarkable chemical stability, even when exposed to acid gases. In addition, it is also stable when exposed to steam and hardly loses its adsorption properties over time. This makes it one of the most promising materials for the capture of CO_2_ from industrial exhaust gases using an energy-efficient steam-based process. Svante Technologies Inc. is commercializing the Rapid Cycle Temperature Swing Adsorption (RCTSA) process for point-source CO_2_ capture (8–20% CO_2_) using CALF-20 as the adsorbent.^7^ The RCTSA process consists of three main steps: adsorption, steam regeneration, and air conditioning.^8^ During adsorption, CALF-20 selectively captures CO_2_ from the flue gas while largely ignoring N_2_. Next, steam regeneration releases adsorbed CO_2_. Finally, the air conditioning step removes excess H_2_O retained during steam regeneration, preparing the adsorbent for the next cycle (Figure 1).^1^

**Figure 1:**
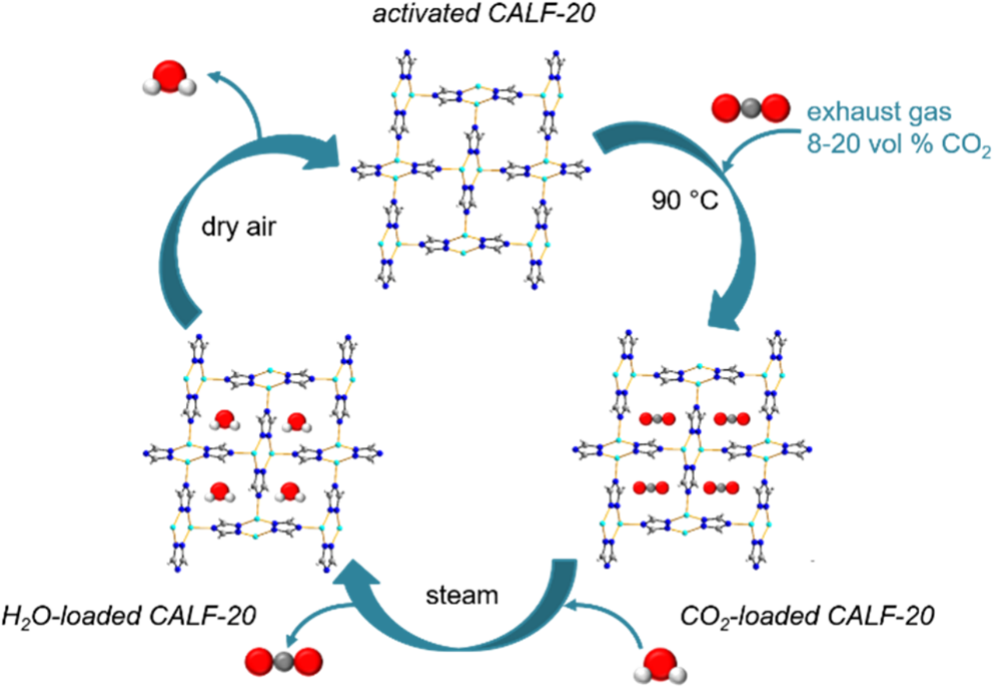
Process for carbon dioxide capture from industrial exhaust gases with subsequent MOF regeneration by using water vapor and drying as envisioned by the Svante Company.^1^

Given its remarkable stability and effectiveness in CO_2_ capture, CALF-20 has been the subject of extensive practical and theoretical studies exploring its sorption capacity^9^ and structural dynamics. During the course of these investigations, it was found that the XRPD pattern of CALF-20 significantly changes depending on the history of the sample,^6,10,11^ which indicates that the MOFs show a very dynamic structural response to external stimuli. Accordingly, a wide variety of CALF-20 polymorphs comprehensively summarized by Drweska et al.^12^ have been reported in the literature. The solution-based synthesis^13^ of CALF-20 leads to the formation of an MOF loaded with *n* solvent molecules, Zn_2_(ox)­(trz)_2_·*n*, also denoted as “α-CALF-20,” which was the first-reported^6^ crystalline phase. When this phase is loaded with CO_2_ after activation, it is denoted as “α-CALF-20-CO_2._”^12^ There are at least three water-loaded CALF-20 polymorphs known: β-CALF-20,^10^ γ-, and τ-CALF-20.^12^ While β-CALF-20 shows the pseudo fourfold coordination sphere of zinc,^8^ in γ-, and τ-CALF-20, the water molecules enter the coordination sphere of the cation and expand it toward sixfold coordination. The incorporation of CO_2_ molecules leads to a slight expansion of the unit cell,^12,14^ whereas water incorporation prompts a contraction.^10,12^ Remarkably, all experimentally known polymorphs of CALF-20 crystallize in a primitive, monoclinic unit cell with the space group *P*2_1_/*c* (14) and similar cell metrics. A systematic *in situ* study on water and carbon dioxide loading and unloading, which has not been performed yet, could furnish a more coherent phase space of CALF-20 and would lead to an overall more consistent picture of all existing polymorphs of this MOF.

To date, there are many works devoted to *in situ* X-ray diffraction of porous materials during gas loading.^15^ Using a custom gas cell, *in situ* studies on CO_2_,^16,17^ noble gases, or CH_4_ sorption^16,18,19^ by both single-crystalline or polycrystalline porous material can be performed. The obtained XRPD data allow us to study the changes in the crystal structure and, in some cases, determine the location of gas molecules in the MOF’s pore.^19,20^ However, for polycrystalline samples, these measurements are usually performed at synchrotron or neutron sources, which are not always available research tools and therefore are not suitable for routine analyses.

In this work, we carried out a systematic study on the CO_2_ and H_2_O loading behavior at different temperatures of CALF-20 using *in situ*, high-resolution laboratory XRPD, leading to an overall more consistent image of the existing polymorphs of this MOF. In addition, we collected single-crystal X-ray diffraction (SCXRD) data on an air-exposed CALF-20 and found that in addition to the coordinated water in τ-CALF-20,^12^ there is an additional water molecule in the pore, which could assist the hydration of CALF-20. Interestingly, the simulated pattern of this phase, θ-CALF-20, matches extremely well with our bulk XRPD pattern presented in this study. A computational study was also included to establish the role of these two independent water molecules in hydrating CALF-20.

## 2 Experimental Section

The CALF-20 powder sample used in this investigation was supplied by BASF. As described in the Supporting Information, CALF-20 powder was synthesized following the method reported in U.S. Patent Application US20240190898A1.^21^

### XRPD Data Collection

XRPD patterns for *in situ* thermal expansion analyses and CO_2_ gas ad- and desorption were collected at room temperature on a laboratory powder diffractometer in Debye–Scherrer geometry (Stadi-P diffractometer (Stoe), Cu–Kα_1_ radiation from primary Ge(111)-Johann-type monochromator, triple array of Mythen 1 K detectors (Dectris)) using a home-built gas loading setup (Supporting Information Figure S1). The samples were filled in 0.5 mm borosilicate glass capillaries (Hilgenberg glass no. 0140), which were attached to a rubber plug (Supporting Information Figure S1b). The capillaries were left open from one side, placed on the holder, and tightly fixed with the metal cap (Supporting Information Figure S1c). The sample holder was connected to the vacuum pump and gas bottles by tubes through a four-way pipe (Supporting Information Figure S1e,f), allowing to control pressure and atmosphere in the capillary.^22^ During the XRPD measurements, the capillaries were rotated in an alternative fashion. Heating and cooling of the capillaries was realized by using a hot and cool air blower (Cobra 700, Oxford cryosystems). Prior to all measurements, CALF-20 was activated by heating to 130 °C under dynamic vacuum (*p* < 1 × 10^–3^ mbar) for 2 h.

#### *In Situ* XRPD on Thermal Expansion under Dynamic Vacuum

After the activation of the MOF, the sample was cooled down under dynamic vacuum (*p* < 1 × 10^–3^ mbar) from 130 °C to −75 °C in 10 and 5 K steps, respectively, applying a cooling rate of 2 K/min. Prior to each measurement, a delay of 5 min was applied to ensure thermal equilibration. For each scan, the data were collected in a 2θ range from 1 to 111°, applying a total scan time of 1 h. After reaching −75 °C, the sample was heated to 130 °C using the same temperature profile and scan parameters.

#### *In Situ* XRPD on CO_2_ Gas Ad- and Desorption

After activation, the MOF was loaded with 970 mbar pure CO_2_ gas (Air Liquide; 99.995%) or artificial CO_2_–air mixture containing 9% CO_2_ (Air Liquide; CO_2_ 9%, O_2_ 18.655%, N_2_ 72.345%) and cooled down from 130 to −75 °C in 10 and 5 K steps, respectively, applying a cooling rate of 2 K/min. Prior to each measurement, a delay of 10 min was applied to ensure thermal and gas loading equilibration. For each scan, the data were collected in a 2θ range from 1 to 111°, applying a total scan time of 1 h.

#### *In Situ* XRPD on H_2_O Vapor Ad- and Desorption

XRPD patterns under controlled relative humidity were collected using a Bruker D8-Advance diffractometer using Cu–Kα_1_ radiation from a Johannson-type Ge(111) monochromator and a Lynx Eye position-sensitive detector (Bruker) equipped with a humidity chamber (Anton Paar). The humidity within the chamber was adjusted by mixing a dry and a water vapor-saturated nitrogen stream. A total flow rate of 500 mL/min was applied, and the temperature of the chamber was constantly kept between 25.0 ± 0.2 °C and 60.0 ± 0.2 °C. All measurements were performed in Bragg–Brentano geometry, using a scan range from 5 to 50° 2 θ, a step size of 0.001°, and a total scan time of 2 h. Prior to the measurements, the MOF was activated in a Schlenk flask at a temperature of 130 °C under dynamic vacuum (*p* < 1 × 10^–3^ mbar). The MOF was at first exposed to low relative humidity (5 or 6% R.H.). The humidity was subsequently increased in 5% R.H. steps until 90% R.H. Before every scan, an equilibration period of 15 min was applied.

### XRPD Data Analyses

The program TOPAS 6.0 ^23^ was used for XRPD data analysis. The collected XRPD patterns of activated CALF-20 were subjected to Rietveld^24^ refinements using the crystal structure model by Lin et al.^6^ (Supporting Information, Figure S2). The instrumental profile was described by the fundamental parameter approach implemented into the TOPAS software,^25^ and the precise lattice parameters were determined by LeBail fits^26^ prior to each crystal structure refinement. For initial localization of the adsorbed carbon dioxide molecules, the data sets recorded at the lowest temperatures, that is, with the highest content of incorporated CO_2_ molecules, were chosen. The gas molecules were modeled by using spherical dummy atoms (having the same number of electrons as a CO_2_ molecule) with large thermal displacement parameters in order to account for the positional disorder. The global optimization method of simulated annealing^27^ was employed to localize the molecules in real space. Water molecules were modeled by oxygen atoms, omitting the hydrogen atoms due to the limits of the XRPD method, and localized as described above. The XRPD patterns of CALF-20 loading states leading to a very large lattice deformation or to phase transitions were indexed *ab initio*.^28^ The crystal structures were determined by applying the global optimization method of simulated annealing. Atoms located in special positions were identified by using an occupancy merge radius of 0.7 Å,^29^ and the organic ligands were constrained by using rigid bodies set up in *z*-matrix notations, which were rotated and translated throughout the unit cells.

## 3 Results and Discussion

We systematically investigated the loading of CO_2_ and water vapor into the CALF-20 MOF by both isobaric and isothermal *in situ* XRPD measurements. In order to decouple the guest-induced breathing behavior from the thermal expansion, we investigated the thermal expansion behavior of the evacuated and activated MOF in detail. The results are presented in the Supporting Information in section 3. Prior to the gas loading experiments, the MOF was activated by heating under a dynamic vacuum to 130 °C (Supporting Information, section 2).

### CO_2_ Gas Ad- and Desorption

The CO_2_ gas ad- and desorption behavior of CALF-20 was investigated by isobaric and isothermal experiments. For the isobaric studies, only cooling experiments were performed, as the anomaly in the thermal expansion of evacuated CALF20 occurring between −10 and 20 °C (Supporting Information Figure S3, b) can be observed reproducibly during cooling, whereas its occurrence shows strong fluctuations during heating.

#### CO_2_ Adsorption and Desorption

Isobaric CO_2_ sorption experiments were carried out at a pressure of 970 mbar using both pure CO_2_ and an artificial CO_2_–air mixture containing 9% CO_2_. After activation of the MOF under dynamic vacuum at 130 °C, the capillary was pressurized with the gas and cooled to −70 °C while recording XRPD patterns. During cooling, the XRPD patterns show changes in the peak intensities, for example, for the 100 reflection at around 11 ° 2θ (for Cu–Kα_1_ radiation) indicating a significant incorporation of CO_2_ molecules into the bulk material and a considerable peak shift (Figure 2a), caused by the corresponding lattice deformation, which will be discussed later. The CALF-20 MOF already starts incorporating CO_2_ into the bulk structure at 130 °C, where we estimated the amount located within the pore with 0.21 mol of CO_2_ per Zn atom for pure CO_2_ and 0.02 mol of CO_2_ per Zn atom for 9% CO_2_ in air (Figure 2b). A decrease in temperature leads to a steep increase in the amount of adsorbed gas for a pure CO_2_ atmosphere, which finally reaches a plateau at −40 °C with 1.10 molecules of CO_2_ incorporated per Zn atom (Figure 2b, black line and symbols). The increase in CO_2_ bulk loading from a 9% CO_2_–air mixture is much smaller (Figure 2b, blue line and symbols) and reaches a maximum at −70 °C with 0.51 molecules CO_2_ incorporated per Zn atom without forming a plateau.

**Figure 2:**
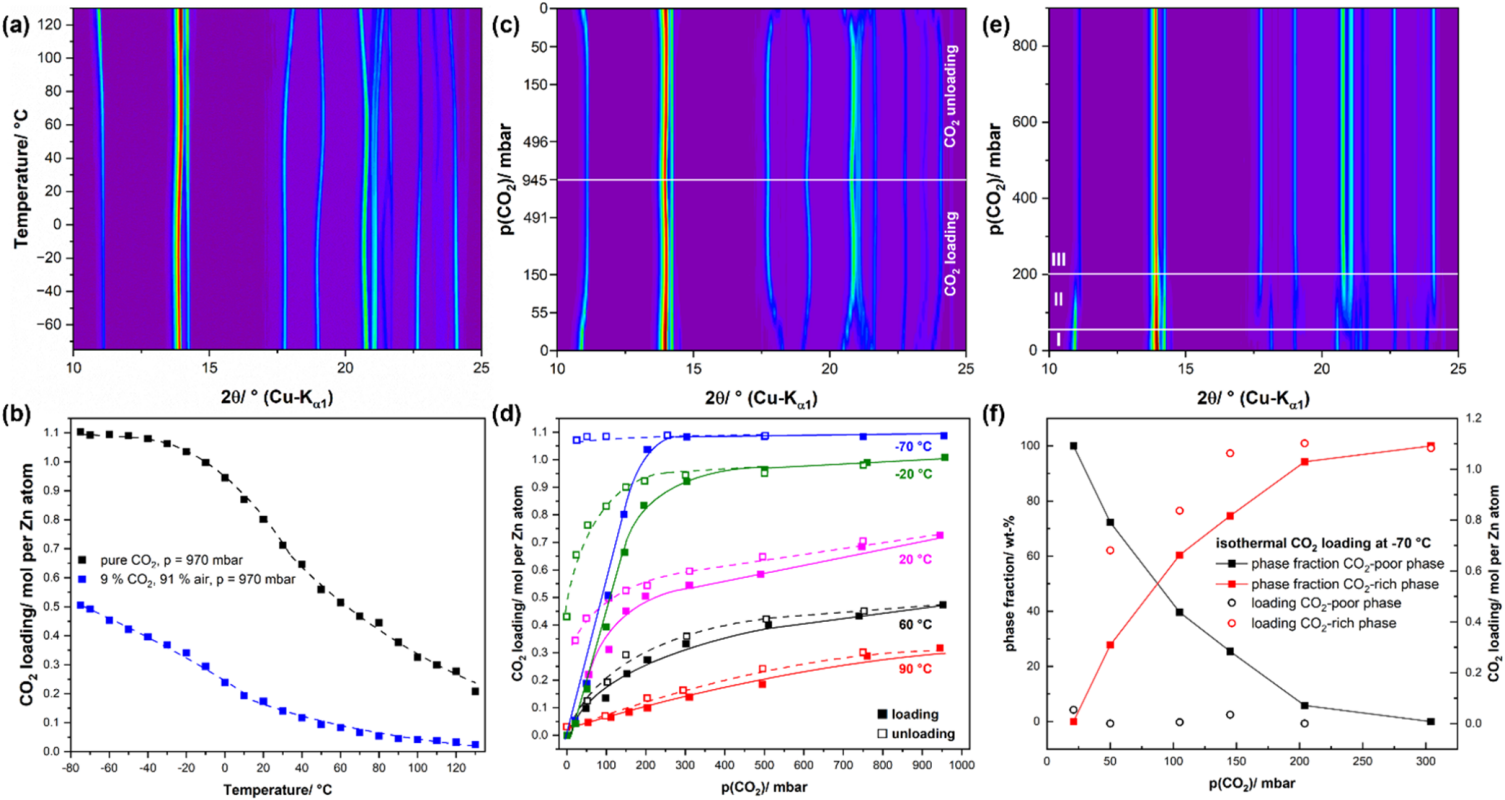
*In situ* XRPD patterns of CALF-20 recorded in pure CO_2_ atmosphere during the (a) isobaric CO_2_-uptake at 970 mbar by cooling from 130 to −75 °C, (b) CO_2_-bulk loading of CALF-20 as obtained from the XRPD data in pure CO_2_ atmosphere (black) and synthetic exhaust gas (blue) during isobaric loading, (c) *in situ* XRPD patterns of CALF-20 during isothermal CO_2_ loading/unloading at 20 °C, (d) CO_2_-bulk loading of CALF-20 during isothermal loading (filled symbols) and unloading (open symbols), (e) *in situ* XRPD patterns of CALF-20 during isothermal CO_2_ loading at −70 °C, I: unloaded phase, II: loaded and unloaded phase, III: loaded, (f) quantitative analyses of the loaded and unloaded phases (closed symbols) and quantification of the adsorbed CO_2_ in the CALF-20 phases during isothermal CO_2_ loading at −70 °C. For all experiments, evacuated and activated CALF-20 was used as a starting material.

For the isothermal CO_2_ sorption experiments, we used a pure CO_2_ atmosphere exclusively but tested both CO_2_ loading and unloading. While increasing the CO_2_ pressure, again, changes in the peak intensities and positions indicate CO_2_ loading (Figure 2c). By reducing the pressure of CO_2_, the diffraction pattern reverts to its original state, but not completely, indicating that some of the incorporated CO_2_ remains within the pores even at <10^–3^ mbar, that is, under dynamic vacuum. A detailed analysis of the diffraction patterns showed that CO_2_ loading increases slowly and linearly with increasing CO_2_ pressure at 90 °C (Figure 2d, red, straight line, and symbols). With decreasing temperature, the initial slope in CO_2_ loading becomes steeper, and the overall loading reaches a plateau. At −70 °C, the plateau is already reached at a CO_2_-pressure of 300 mbar (Figure 2d, blue, straight line). Reducing the CO_2_-pressure leads to increasing and eventually complete unloading at least for temperatures ≥60 °C (Figure 2d, red and black, dashed lines). At lower temperatures, an increasing amount of CO_2_ remains within the pores, even under dynamic vacuum (magenta, green, and blue, dashed lines) and at −70 °C, almost the entire amount of incorporated CO_2_ remains within the bulk. A close investigation of the XRPD patterns recorded during the CO_2_ sorption at −70 °C reveals additional interesting phenomena. Initially (0 mbar) and at low CO_2_ pressure (21 mbar), one crystalline, unloaded CALF-20 phase is present (Figure 2e,I). By increasing the CO_2_-pressure (50 mbar), two distinct crystalline phases of CALF-20 become apparent (Figure 2e,II). By further increasing the CO_2_-pressure, the sample gradually shifts toward a single-phase state (III). The patterns recorded during the intermediate stage of CO_2_-loading (50–204 mbar) could indeed be refined using two identical CALF-20 structures with distinct, individual lattice parameters and pore filling states (Supporting Information, Figure S5). For all patterns, one of the CALF-20 phases refined with almost empty pores (Figure 2f, black open circles), that is, the “unloaded phase,” decreases, whereas the CO_2_ loading of the second phase, the “loaded phase,” was observed to increase with increasing CO_2_-pressure (red circles). Accordingly, during CO_2_ loading, the phase fraction of the “loaded phase” increased, whereas the “unloaded phase” is consumed (Figure 2f, black and red lines), which may point to a core–shell mechanism or to particles of different sizes transforming at different rates. At 300 mbar of CO_2_ pressure, the conversion is finally completed.

#### CO_2_ Breathing Behavior

To show a more detailed picture of the CO_2_-breathing behavior of CALF-20, we present the evolution of the absolute difference of the lattice parameters of the CO_2_-loaded MOF from the lattice parameters of the activated and evacuated MOF at the respective temperatures (Supporting Information, Figure S3b) with increasing CO_2_ content in Figure 3a. Initial CO_2_ loading into CALF-20 leads to a significant expansion of the *b*-axis, analogous to the observations by Drweska et al.,^12^ whereas the unit cell contracts moderately in *a-* and [001] directions. This corresponds to a distortion of the nearly rectangular, staggered channel running in the [1̅01̅] direction into a more diamond-like shape (Figure 3b, green and orange arrows). Starting at a pore filling state between 0.5 and 0.7 mol CO_2_ per Zn ion, the breathing behavior changes: the unit cell slightly contracts along the *b*-axis and significantly expands in [001] direction, whereas there is hardly any change in the *a*-direction (Figure 3a). Hence, a large population of the pore channels by CO_2_ molecules leads to a decreasing diamond-like distortion of the pore channels and shifts them into a more eclipsed state (Figure 3b). Overall, CO_2_ loading into CALF-20 leads to an increase in the unit cell volume: 733.9 Å^3^ (at −20 °C, under vacuum) vs 741.3 Å^3^ (at −20 °C, 1.04 mol CO_2_ per Zn atom). In addition, the thermal expansion behavior is altered, whereas under vacuum evacuated CALF-20 shows a negative volume expansion: 734.6 Å^3^ (at −75 °C) vs 733.9 Å^3^ (at −20 °C) (Supporting Information, Figure S3b and Table S1) and CO_2_-loaded CALF-20 exhibits positive thermal expansion: 738.4 Å^3^ (at −75 °C, 1.10 mol CO_2_ per Zn atom) vs 741.3 Å^3^ (at −20 °C, 1.04 mol CO_2_ per Zn atom) (Supporting Information, Table S3). We tentatively attribute the positive thermal volume expansion occurring between −75 and −20 °C, that is, within the plateau of CO_2_ loading (Figure 2b), to a reduction in thermal motion of the guest molecules.

**Figure 3:**
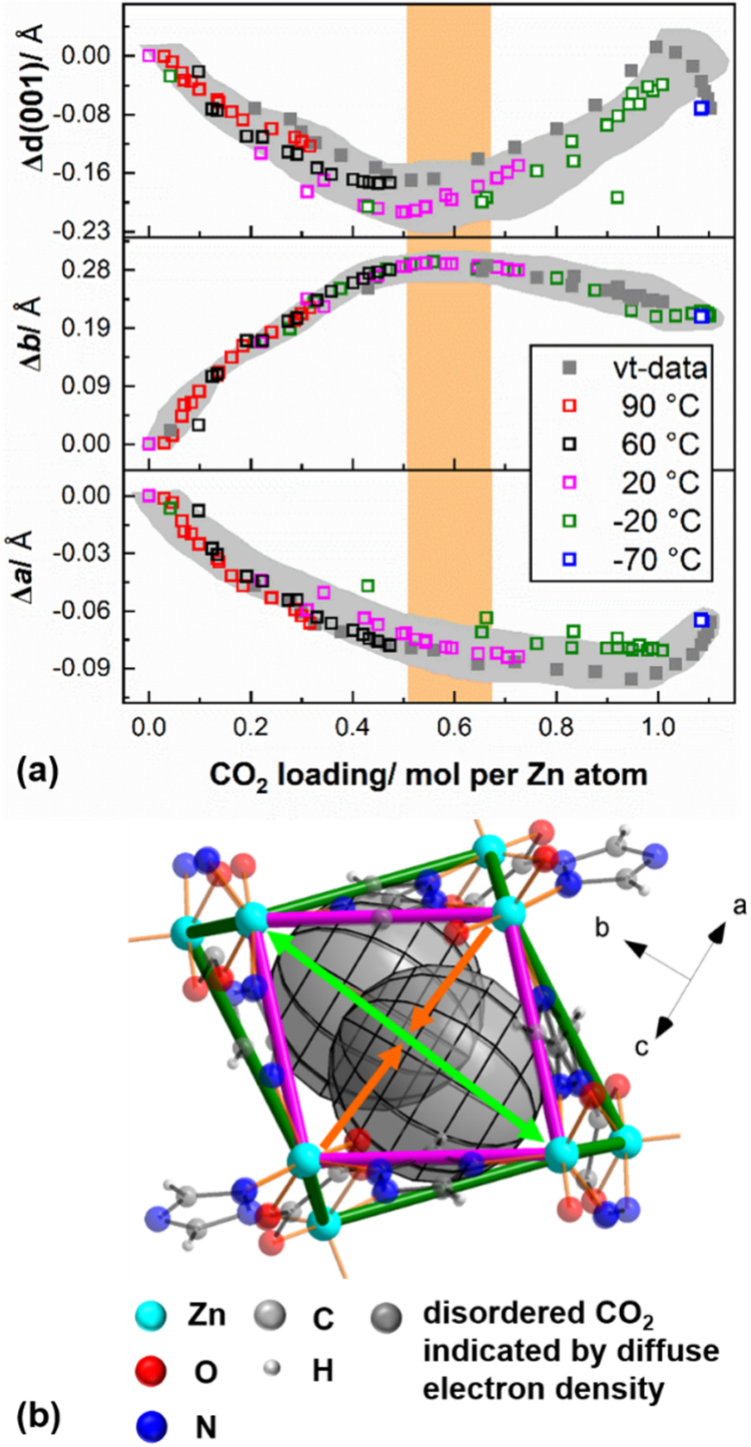
(a) Relative change in lattice parameters upon isothermal and isobaric (variable temperature (vt)-data) CO_2_ adsorption of CALF-20 corrected by thermal expansion (Supporting Information, Figure S3). The trend in change of lattice parameters is highlighted by the gray background. The change in breathing behavior is indicated by the orange background. The errors in lattice parameters correspond to the size of the symbols. (b) View of a pore of CALF-20 in the [**1̅01̅**] direction. The staggered pore is indicated by magenta and green bonds, and orange and light green arrows indicate the directions of pore deformation upon CO_2_ loading.

### H_2_O Vapor Ad- and Desorption

The water adsorption and desorption behavior of CALF-20 was systematically investigated by isothermal *in situ* XRPD measurements. During these experiments, the activated MOF was exposed to a dry nitrogen stream, and the relative humidity was iteratively increased from 5 to 90%. At 25 °C, a phase transition can be observed between 20 and 30% R.H. (Figure 4,a). Like evacuated and activated CALF-20 (Supporting Information, Table S2), both phases crystallize in a primitive, monoclinic lattice with space group *P*2_1_/*c* (14), having closely related cell metrics (Supporting Information, Figure S6, inset). The transition from water-loaded CALF-20 phase I to phase II leads to an anisotropic deformation of the unit cell, whereas the *a-* and *c-*axes expand by ≈0.3 and ≈0.7 Å, respectively, the *b*-axis shrinks by ≈1.7 Å, which is clearly indicated by the downshift of the 100 peak and the upshift of the 011 and 110 reflections (Supporting Information, Figure S6). Rietveld refinements (Supporting Information, Figure S7) revealed that both phases contain water in the bulk. Already at 6% R.H., we detected almost 0.4 molecules of water per zinc cation in the pores (Figure 4c, black squares). Until 50% R.H., the amount of incorporated water rapidly increases to ≈1.65 molecules of water per zinc cation. Further increase in relative humidity only leads to a slight, additional accumulation of pore water, which finally reaches around 1.8 molecules of water per zinc cation. The water incorporation is fully reversible, as decreasing the relative humidity leads to a release of pore water (Supporting Information, Figure S8). There is a slight hysteresis behavior between 40 and 20% R.H., which is attributed to the slow transition from water-loaded CALF-20 phase II to phase I (Supporting Information, Figure S8, gray background). *In situ* XRPD measurements at 55 °C reveal that the phase transition from water-loaded CALF-20 phase I to phase II is inhibited by increasing the temperature (Figure 4b). Although the reflections attributed to phase I show pronounced shifting, in particular at high relative humidity, no additional peaks appear. Systematic investigation reveals that the transition from phase I to phase II gradually shifts toward higher relative humidity with increasing temperature (Figure 4d). Starting at 45 °C, the phase transition is not completed upon reaching 90% R.H. (Figure 4d, blue line and squares), and the maximum conversion rate iteratively drops with increasing temperature. Interestingly, an increase in the temperature does not affect the amount of water that is incorporated into the structure of CALF-20 at a given relative humidity (Figure 4,c, gray area), despite the fact that the phase transition is suppressed and that an increase in temperature at a constant relative humidity corresponds to an increase in water vapor partial pressure. The water-breathing behavior of water-loaded CALF-20 phases I and II is illustrated in Figure 4e,f. The breathing behavior of phase I changes upon water loading: initially, the incorporation of water molecules leads to a significant contraction of the unit cell in the [001] direction, whereas it slightly expands along both the *a*- and *b*-axes. Starting at a water loading between 1.0 and 1.2 molecules per zinc cation, the expansion of the *a*-axis becomes steeper, and the cell also starts expanding in the [001] direction, whereas the *b*-axis shows significant contraction (Figure 4e). Overall, the volume of phase I decreases by the incorporation of water molecules. Phase II only occurs at a water loading of ≥1.1 molecules per zinc cation (Figure 4f). In contrast to phase I, phase II shows volume expansion upon water incorporation and does not exhibit a change in the breathing behavior. An incorporation of water molecules into phase II leads to an expansion along both the *a*- and *b*-axes, whereas the unit cell contracts in the [001] directions. Phase II shows a significant thermal expansion (Figure 4f, top, black, and red squares), which is mainly driven by the expansion of the *b*-axis. In contrast, phase I does not show a significant thermal expansion (Figure 4e). This could be the reason why an increasing temperature inhibits the transformation from phase I to phase II since at higher temperatures the packing of phase I is more efficient than for phase II.

**Figure 4:**
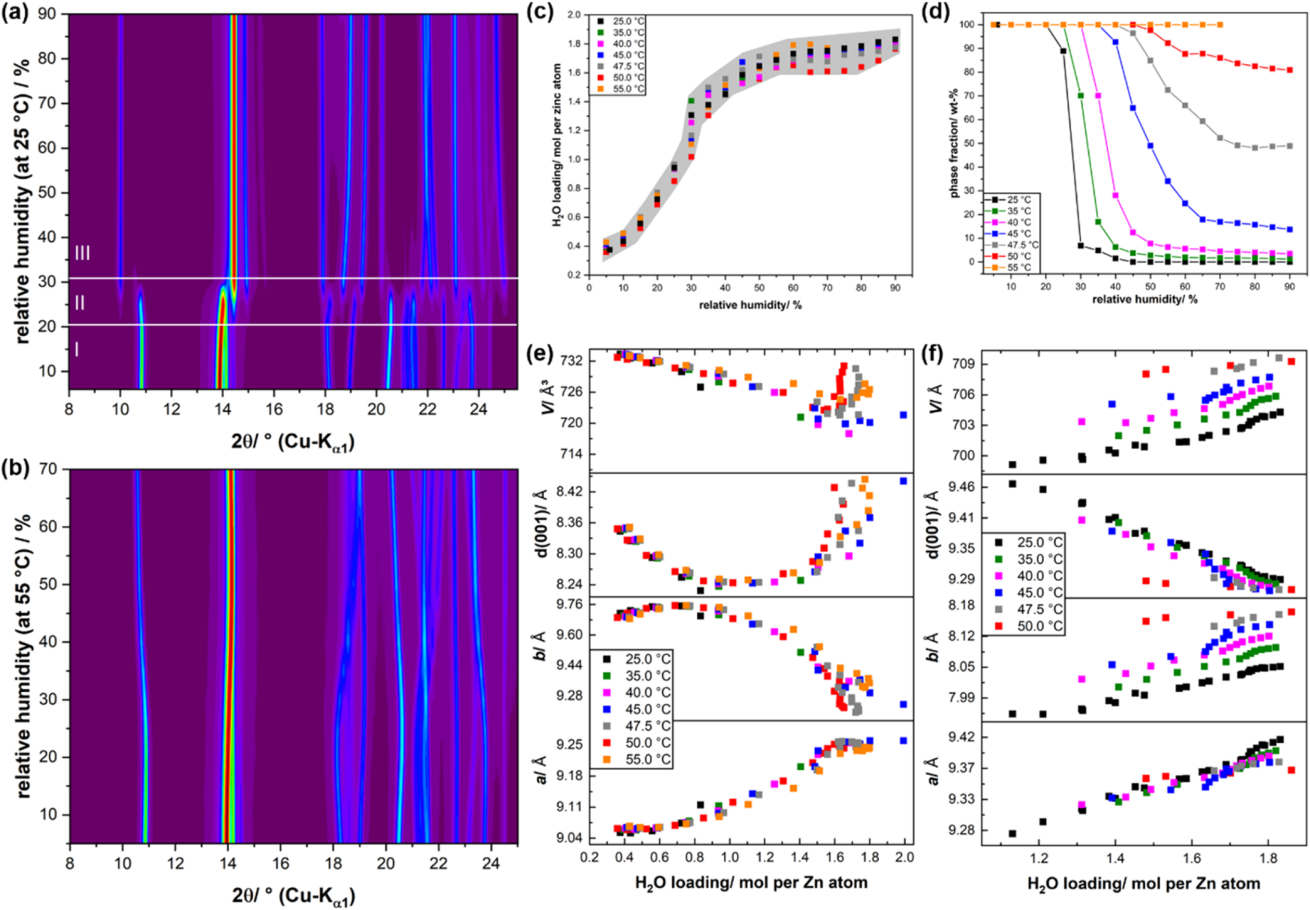
*In situ* XRPD patterns of CALF-20 recorded under isothermal conditions at (a) 25 °C (with I = water-loaded phase I, II = water-loaded phases I + II, III = water-loaded phase II) and at (b) 55 °C using variable relative humidity, (c) water loading of CALF-20 as obtained from the XRPD data as a function of temperature and relative humidity, the gray background highlights the trend, (d) phase fraction of CALF-20 water-loaded phase I as a function of temperature and relative humidity, the phase fraction of water-loaded phase II amounts to 100 wt %—phase fraction (water-loaded phase I); lattice parameters of (e) CALF-20 water-loaded phase I and (f) phase II as a function of temperature and water loading. For all experiments, evacuated and activated CALF-20 was used as a starting material.

### Implications from Guest-MOF-Framework Interactions

The high crystallinity of the investigated CALF-20 samples, as well as the quality of the diffraction data, enabled high-quality Rietveld refinements of the *in situ* data during both CO_2_ and H_2_O loading. This enables an atomistic view of the adsorption process that goes beyond the lattice deformation indicated by the shift of the reflection positions. In its activated state, that is, with pores being empty, the zinc cation exhibits a fivefold coordination (Figure 5a). Full CO_2_ loading of up to 1.1 molecules per zinc cation does not affect this coordination sphere. Within the pores, the carbon dioxide molecules are positionally disordered and appear as diffuse clouds of electron density (Figure 5b, gray, transparent globes). This confirms previous experimental and theoretical findings.^12,14^ The barycenter of this diffuse electron density is located off-center of the pore, with the pore center being the symmetry center at the 2d position. An increasing CO_2_ loading shifts the diffuse electron density to be more off-center of the pores. We could correlate this with the change in CO_2_ breathing behavior occurring between 0.5 and 0.7 mol CO_2_ per Zn ion (Figure 3a, orange background). An exact localization of the guest molecules from XRPD data is impossible, as the positional disorder of the CO_2_ molecules is too pronounced. The change in CO_2_-breathing behavior may point to the existence of an α-CALF-20·*n*CO_2_ phase and an α′-CALF-20·*n*CO_2_ phase.

**Figure 5:**
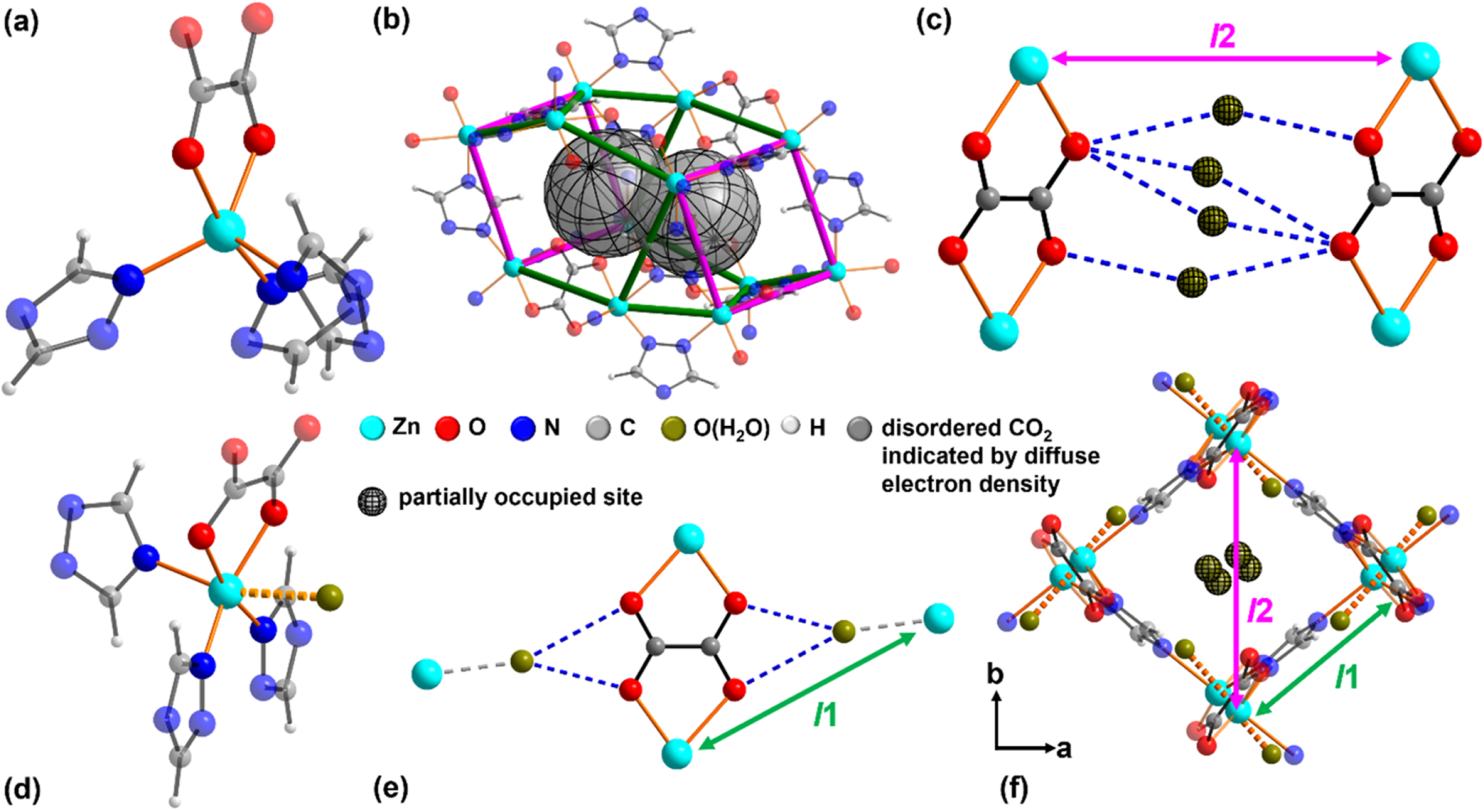
(a) Zn coordination in activated and evacuated CALF-20, (b) CALF-20 pore with pore windows highlighted by magenta lines including off-center locations of positionally disordered CO_2_ molecules indicated by diffuse electron density (gray, transparent globe spheres), (c) interactions of noncoordinating water molecules with oxalate ligands in hydrated CALF-20, (d) Zn-coordination in hydrated CALF-20, (e) interactions of coordinating water molecules with oxalate ligands in hydrated CALF-20, and (f) top-view on the pore of CALF-20 with characteristic lengths that change upon gas loading (Table 1) indicated as *l*1 and *l*2.

The incorporation of water into CALF-20 leads in its initial stage, that is, for water-loaded CALF-20 phase I with a water loading of <1.0 molecules per zinc cation, to the appearance of a narrow rod of diffuse electron density in the center of the pores running parallel to the Zn^2+^–(C_2_O_4_)^2–^–Zn^2+^ bridges indicating the presence of positionally disordered water molecules (Figure 5c, green globes). Comparing this to the literature data, this phase reassembles “a slightly hydrated form of α-CALF-20, without noticeable structure alteration.”^10^ The comparatively short distance between these water molecules and oxalate anions (<3 Å) points to the presence of hydrogen bonds (blue, dashed bond). Upon increased water loading, additional molecules can be localized, entering the coordination sphere of zinc (Figure 5d, green ball). As a result, the zinc cation adopts a distorted octahedral coordination as described for “τ-” and “γ-CALF-20·*n*H_2_O.”^12^ This water molecule is also located in the proximity of the oxalate anion, which potentially enables the formation of hydrogen bonds (Figure 5e, blue, dashed bond). At low temperatures (≤40 °C), this process is associated with the transition from water-loaded CALF-20 phase I to phase II. An extension of the fivefold coordination sphere of zinc to a distorted octahedral coordination also occurs in phase I, when the water loading increases by one molecule per zinc cation at elevated temperatures (≥40 °C). This is associated with the observed change in the breathing behavior of phase I at a water loading between 1.0 and 1.2 molecules per zinc cation (Figure 4e). Thus, on an atomistic scale, water-loaded CALF-20 phases I and II appear to be identical at high water loading, with both phases containing disordered water molecules in the pore channels and additional water, which is coordinated to the zinc cations.

A comparison of the unit cell volumes of evacuated and pore-filled CALF-20 from the Rietveld refinement reveals implications for competing sorption of CO_2_ and water vapor. In relation to the unit cell volume of activated CALF-20, the incorporation of CO_2_ molecules leads to an increase in the unit cell volume from 733.6 to 737.6 Å^3^ (Table 1). On the contrary, the uptake of water leads to a decrease in unit cell volume to 732.6 Å^3^ for 0.43 molecules per zinc cation and to 701.1 Å^3^ for 1.59 molecules per zinc cation. This contraction is mainly driven by the shrinking of the pore diagonal (Figure 5c,f and *l*2) and is most likely caused by hydrogen bridges between positionally disordered water molecules in the center of the pores and oxalate ligands (Figure 5e). The length of the pore wall (Figure 5e,f and *l*1) is not affected by any incorporation of host molecules. Water is able to expel CO_2_ from CALF-20, as the uptake of water molecules leads to a smaller resulting unit cell volume and therefore to a more efficient atom packing, which is also more stable than the packing observed in the CO_2_-loaded CALF-20. To add further significance to the characterization of this hydrated phase, we resorted to single-crystal XRD. These additional water molecules in the pore channels are evidently located in the θ-CALF-20 crystal, as confirmed by SCXRD analysis (Figures 6 and S9a). Interestingly, the θ-CALF-20 phase was obtained by exposing activated α-CALF-20 crystals to ambient conditions of 25–27 °C, in ambient air for a period of 14 days. Nevertheless, as the thermal expansion behavior differs (Figure 4e,f), we think that the two forms of CALF-20 are two distinct phases, indeed. Phase I seems to be closer to “α-CALF-20·*n*H_2_O,”^10^ whereas the assignment of “τ-” and “γ-CALF-20·*n*H_2_O” is impossible, as there is no report on the thermal expansion behavior of these phases.^12^ Notably, exposing α-CALF-20 crystals to ambient conditions induced an SC-to-SC phase transition, resulting in the formation of θ-CALF-20 (Figures 6 and S9a). In addition to the significant changes in the lattice parameters and reduction in unit cell volume (the crystallographic axes length changes: *a*-axis by 4.7%, *b*-axis by 17.4%, and *c*-axis by 5.6% leading to a volume change of 4.96%, and the β angle decreases by 4.96%, Tables S4 and S5). When compared with α-CALF-20, the key difference between the two phases lies in the zinc coordination sphere. While both phases share the same monoclinic space group *P*2_1_/*c* (14), they adopt two distinct Zn­(II) coordination geometries. In α-CALF-20, the Zn^2+^ cation is five-coordinated with a distorted trigonal bipyramidal geometry, whereas in θ-CALF-20, the zinc center has a distorted pseudo-octahedral geometry with a ligated H_2_O molecule to Zn^2+^ cation (Zn–O_W_ distance = 2.464 Å; O_W_ occupancy 72%). A thorough examination of crystallographic data provided for reported τ-CALF-20 ^12^ suggests that our θ-CALF-20 crystal is remarkably similar to this phase. The only differences between these two crystal phases are, first, the coordinated H_2_O occupancy factor and, second, the identified additional free H_2_O molecule in the pore within a distance of 2.846 Å from the coordinated H_2_O (Figures 5 and S9). The electron density was reliably assigned to this free H_2_O molecule, which was 77% occupied.

**Table 1:** Overview of Structural Parameters Including Characteristic Lengths, *l*1 and *l*2 along the Pores (Figure 5f) as a Function of the Host Molecule

status at 25 °C	loading/mol guest molecule per Zn atom	*l*1/Å	*l*2/Å	*V*/Å^3^
evacuated	0	6.05	9.34	733.6
CO_2_ loaded	0.80	6.06	9.30	737.6
H_2_O-loaded phase I	0.43	6.06	9.32	732.6
H_2_O-loaded phase II	1.59	6.10	8.01	701.1

**Figure 6:**
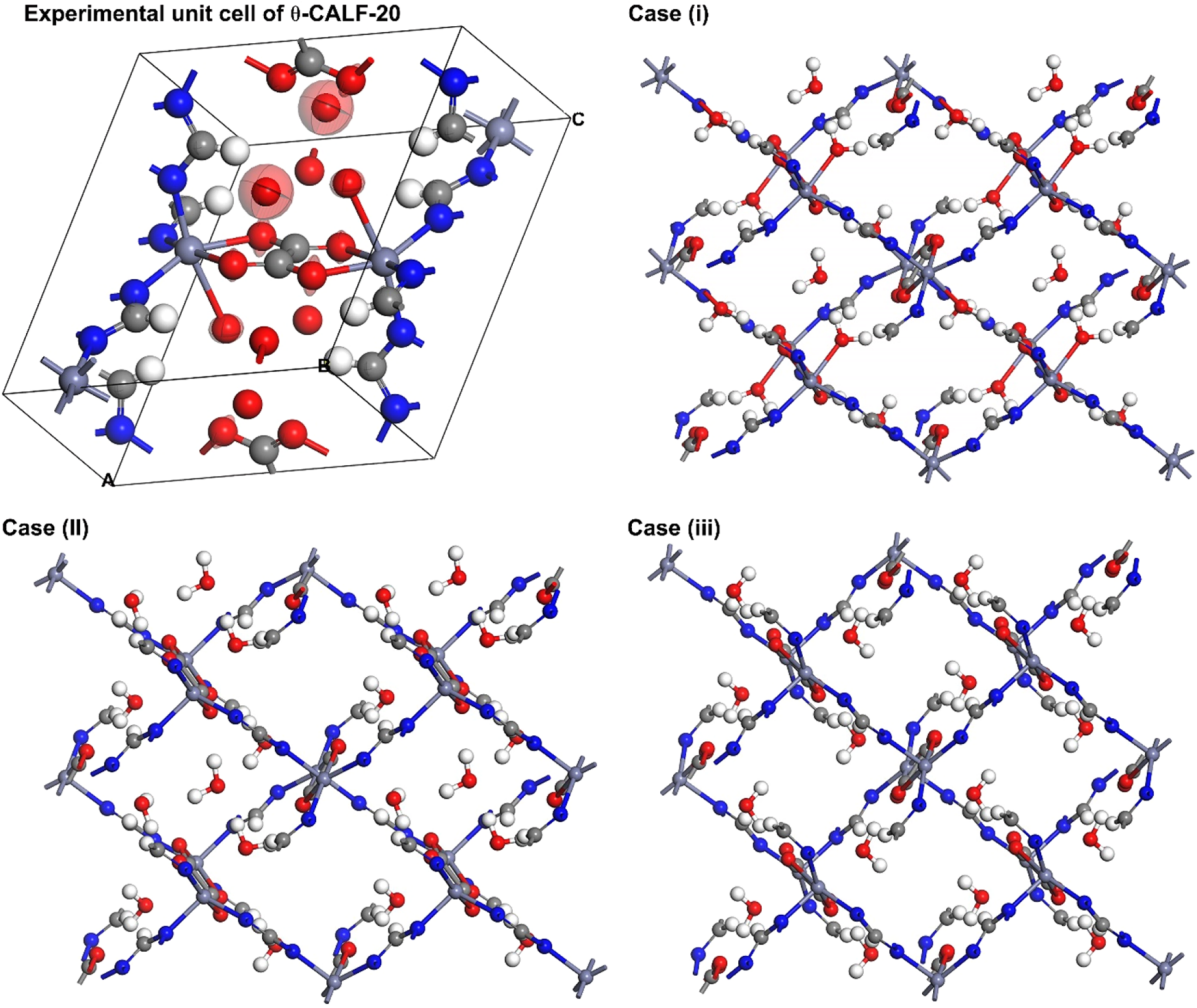
Experimental and modeled structures of θ-CALF-20 showing the cases (i), (ii), and (iii) described in the text. Note: In the experimental structure, the thermal ellipsoids are at 50% probability, and the hydrogens of the water molecules are not shown.

To investigate and understand the impact of coordinated water on θ-CALF-20 stability, first-principles simulations were conducted on the SCXRD-resolved structure. Binding energy (*E*_b_) calculations reveal the influence of coordinated water on the placement of free water. Understanding these interactions is essential for characterizing the system’s stability and hydrophilicity. Three scenarios were analyzed: (i) the binding energy of free water in the presence of coordinated water; (ii) the binding energy of coordinated water with fixed free water in its experimentally determined position; (iii) a final scenario examined CALF-20 without free water, allowing the coordinated water to move freely (Figure 6). In the first scenario, the results aligned with experimental data, showing that coordinated water directs the incoming free water to its position. Simulations indicated that coordinated water forms hydrogen-bonding chains between water molecules and oxalate, enhancing both binding energy (*E*_b_ = −68.299 kJ/mol) and hydrophilicity (Supporting Information, Figures S10 and S11). In the second scenario, the coordinated water rearranged to maximize hydrogen bonding with free water and oxalate, further increasing the binding energy (*E*_b_ = −69.261 kJ/mol). Ultimately, the last check confirmed that removing free water allowed coordinated water to form a stable hydrogen bond with oxalate, suggesting that water is not permanently bound and seeks an energetically favorable configuration. This makes θ-CALF-20 with coordinated water a metastable phase, likely stabilized by kinetic factors. The findings clearly show that the structure with coordinated water is a metastable phase, while CALF-20, which lacks coordinated water, is a significantly more stable low-energy phase—likely the α-CALF-20 phase. Coordinated water enhances the system’s hydrophilicity by facilitating hydrogen bonding with oxalate, highlighting its vital role in organizing free water and thereby enhancing the hydrophilicity of CALF-20. It would be interesting to design a six-coordinated, nonhydrated CALF-20 MOF for superior hydrophobicity. For immediate reference, the CO_2_ and H_2_O adsorption–desorption isotherms of CALF-20 collected at multiple temperatures are provided in the Supporting Information, Figures S12 and S13.

### Implications for Flue Gas Capture

Even though the presented investigation on CO_2_ and H_2_O adsorption into polycrystalline bulk CALF-20 does not entirely mimic all the conditions of the industrial process (Figure 1), some conclusions can still be derived. First, we used polycrystalline CALF-20 powder for all investigations, and the industrial process is also based on polycrystalline powders and not on single crystals. The material retained its crystallinity even after multiple cycles of carbon dioxide or water adsorption and subsequent activation by heating under a dynamic vacuum. This establishes the remarkable stability of this MOF, even under demanding operation conditions. Furthermore, it could be demonstrated that the bulk loading of CO_2_ can be significantly increased if the exhaust gas sorption process step is performed at lower temperatures. A reduction in temperature from 90 to 80 °C leads to an increase in the bulk adsorption by 20%, that is, from 0.045 mol per Zn atom to 0.054 mol per Zn atom for 9% CO_2_ in air (Figure 2b, blue symbols). Understanding the structural behavior under the CO_2_/H_2_O binary environment is valuable for the flue gas sorbent design. In this study, we find that water is able to replace carbon dioxide since its uptake leads to a loading dependent reduction of the unit cell volume and to an expansion of the coordination sphere of zinc, which is energetically reversible (Figures S10 and S11) much more favorable than an expanded cell, with pores populated by disorder CO_2_ molecules (Figures 4e and 5d). Moreover, the observed volume expansion by CO_2_ adsorption and contraction by H_2_O uptakes, however, imply that the CALF-20 adsorber bed is exposed to significant mechanical stress during the flue gas capture process. This study demonstrates that the molecular-level CO_2_ adsorption and hydration–dehydration-related structural changes of CALF-20 are reversible, and the bulk material is accommodative of the volume changes. Also, understanding these structural dynamics is crucial for optimizing adsorption selectivity. The observed crystallite level stabilities augment well with the single-crystal studies and industrial-level demonstrations—Svante has successfully demonstrated at scale in its industrial CO_2_ capture plant using engineered structured CALF-20 beds and optimized steam-assisted cyclic process conditions that such volume changes have not compromised the performance and long-term stability of the CALF-20 adsorber [*Science paper*].

## 4 Conclusions

The CO_2_ and H_2_O sorption behavior of the MOF CALF-20 was investigated systematically by *in situ* XRPD. The MOF can incorporate more than 1.1 molecules of CO_2_ per zinc atom in its pore, which is located off-center. A CO_2_ uptake initially distorts the rectangular pore into a diamond shape. Upon further CO_2_ incorporation, the breathing behavior changes, and the pore becomes more rectangular again. At low temperatures (−70 °C), uptake of CO_2_ initially leads to a two-phase mixture of loaded and unloaded CALF-20, with the latter one being gradually consumed under increased CO_2_ pressure. The gas is bound strongly to the framework and cannot even be removed by a dynamic vacuum. During water uptake of CALF-20, two distinct hydrated phases can be identified: water-loaded CALF-20 phase I and phase II, which confirms previous reports.^10,12^ Phase II exhibits pronounced positive thermal volume expansion, whereas phase I does not expand upon heating. In consequence, the transition from phase I to phase II is inhibited by elevated temperatures. The overall water loading capacity was found to be independent of temperature. Water is incorporated both in the center of the pores and in the coordination sphere of zinc. Potential hydrogen bonds between incorporated water and oxalate ligands lead to a volume contraction of CALF-20, which is most likely the driving force for the extrusion of CO_2_ by water. The θ-CALF-20 identified through SCXRD, and the modeling studies reveal that the coordinated water containing phases of CALF-20 are metastable kinetic phases. These phases tend to attract additional water into the pores. Overall, many different forms of unloaded and water or carbon dioxide-loaded CALF-20 have been reported so far (Supporting Information, Table S6). Considering the unit cell metrics, the pore and the coordination sphere of zinc, both α-CALF-20-activated^12^ (from SCXRD) and evacuated and activated polycrystalline CALF-20 (from XRPD, this work); α-CALF-20-CO_2_ ^12^ (from SCXRD) and CO_2_-loaded polycrystalline CALF-20 (from XRPD, this work); γ-^12^ (from SCXRD), τ-^12^ (from SCXRD), θ-CALF-20 (from SCXRD, this work), and polycrystalline water-loaded CALF-20 phase II (from XRPD, this work) exhibit large similarities. However, as it was demonstrated for water-loaded CALF-20 phases I and II, these criteria are insufficient for distinguishing polymorphs of CALF-20, as differences in thermal expansion behavior (Figure 5f) or breathing behavior may be more indicative. This requires systematic *in situ* studies. After all, this work demonstrates that recent advances in XRPD hard- and software enable detailed investigation of the loading and breathing behavior of a crystalline MOF using laboratory equipment, turning *in situ* studies of gas uptake into routine investigations.

## Supplementary Material



## Notes

Detailed information on the synthesis of polycrystalline CALF-20 bulk powders and single crystals; detailed information on collection of single-crystal diffraction data; detailed information on home-built gas loading setup, the characterization and activation of as-synthesized CALF-20 bulk power; detailed information on the vacuum expansion behavior of CALF-20; additional figures on CO_2_ and H_2_O sorption behavior; crystallographic details of hydrated CALF-20 single crystals including a CIF file; details on the computational method; CALF-20 water and CO_2_ sorption isotherms; a comparison of the crystallographic data of the CALF-20 forms, which have been published so far; CIF files for evacuated and activated polycrystalline CALF-20, CO_2_-loaded polycrystalline CALF-20, polycrystalline water-loaded CALF-20 phase I and phase II (from XRPD) (PDF)

## Abbreviations

XRPD: X-ray powder diffraction
SCXRD: single-crystal X-ray diffraction
vt: variable temperature
