# Supplementary material for: CO2 and H2O Sorption Induced Bulk-Phase Changes of CALF-20 Captured Using In Situ Laboratory X‑ray Powder Diffraction
Source: J Am Chem Soc. 2025 Jul 11;147(29):25662–71. doi: 10.1021/jacs.5c06866 (PMC12291430; doi:10.1021/jacs.5c06866)
Supplement: Supplementary file 1 [file ja5c06866_si_001.pdf]

-Supporting Information-

CO_2 and H_2O sorption induced bulk-phase changes of CALF-20 captured using *in situ* laboratory X-ray powder diffraction

Sebastian Bette[a, *], Anastasiia Sleptzova[a], Bettina V. Lotsch[a,b], Robert E. Dinnebier[a], Stefan Marx[c], Mahsa Loloei[d], Adebayo A. Adeleke[d], Nima Masoumifard[d], Ramanathan Vaidhyanathan[d,*]

[a]Max Planck Institute for Solid state Researach, Heisenbergstraße 1, 70569 Stuttgart, Germany, e-mail: S.Bette@fkf.mpg.de

[b] Department of Chemistry, Ludwig-Maximilians-Universität (LMU), Butenandtstrasse 5-13, 81377 Munich, Germany

[c] BASF SE, Carl-Bosch-Strasse 38, 67056 Ludwigshafen, Germany

[d] Svante Inc., 8800 Glenlyon Pkwy, Burnaby, BC V5J 5K3, Canada., [*] e-mail: vramanathan@svanteinc.com

1. Synthesis methods

Synthesis of CALF-20 polycrystalline bulk phase as powders

As described in US20240190898A1, CALF-20 was synthesized under atmospheric reflux conditions at 100°C. In a typical synthesis, zinc carbonate basic (336 g, 3 mol) was dispersed in deionized water (600 mL) in a 5L three-neck round-bottom flask equipped with an overhead stirrer, thermocouple, condenser, and heating mantle. Oxalic acid dihydrate (190.6 g, 1.507 mol) was gradually added over 8 minutes under controlled agitation, triggering CO_2 release. After 30 minutes of further stirring, a solution of 1,2,4-triazole (208.9 g, 3 mol) in 400 mL of deionized water was introduced, followed by additional mixing to ensure complete CO_2 evolution. The reaction mixture was then heated to reflux (100°C) under continuous stirring (250 rpm) for 1 hour. After cooling with distilled water, the suspension was filtered, and the solid product was washed until the filtrate conductivity dropped below 100 µS/cm. Finally, the material was dried at 110°C for 20 hours.

Synthesis of CALF-20 single crystals

General Remarks

All reagents and solvents were used as received from commercial sources without further purification. Ethanol (reagent grade) and Type I Ultrapure water were employed as solvents.

Synthesis

CALF-20 crystals were synthesized by in-situ decomposition of 2,5-Dihydroxy-1,4-benzoquinone to oxalate. In brief, 0.36 mmol 2,5-dihydroxy-1,4-benzoquinone (0.050 g) was dissolved in 6mL of 50% reagent EtOH and added dropwise into a 6mL 50% EtOH solution of 0.61 mmol $Zn(NO_3)_2{\cdot}6H_2O$ (0.181 g), and 0.73 mmol 1H-1,2,4-Triazole (0.050 g). The resulting deep purple mixture was stirred at room temperature for five minutes, followed by gravity filtration. Then 6mL of clear deep pink filtrate was transferred into a 23mL Teflon-lined autoclave. The autoclave was placed inside a convection oven at 180˚C for 48h. A 2h heating ramp to 180˚C was applied, followed by a 12h cooling ramp to 25˚C.

Single Crystal X-ray Diffraction (SC-XRD) for colorless block-shaped crystals of CALF-20 in mother liquor was collected on a Bruker D8 VENTURE. This solved CALF-20 structure is similar to the previously published phase of α-CALF-20.[1]

Conversion of α-CALF-20 to θ-CALF-20 in open-air

To investigate any changes in α-CALF-20 crystal structure or morphology under ambient conditions, crystals were exposed to ambient air (room temperature). This approach allowed for the observation of a single-crystal-to-single-crystal (SC-to-SC) transition. In a typical experiment, the α-CALF-20 crystals were isolated from mother liquor by vacuum filtration, rinsed with MeOH and dried in ambient air for 15 minutes. The θ-CALF-20 phase was obtained after keeping the dried α-CALF-20 crystals in an unsealed vial for a period of fourteen days at ~25-27˚C. The structure of this novel phase was solved by SC-XRD.

2. Single Crystal X-ray diffraction data collection of θ-CALF-20:

SC-XRD data for this study were collected using a Bruker D8 VENTURE diffractometer equipped with Cu IμS DIAMOND II microfocus Kα source (λ = 1.54178 Å). The instrument was operated at 50 kV and 1.2 mA with a PHOTON 100 detector at 296 K for θ-CALF-20. Crystals were transferred to a crystal oil and mounted on MicroLoops LD™ loop with thin polyimide tips. Data collection and data reduction, and multi-scan absorption correction were performed using the APEX5 software suite, solved with the Bruker SHELXTL software package using direct methods and refined by full-matrix least-squares techniques against F^2 with the SHELXL program package. Non-hydrogen atoms were refined anisotropically. Hydrogen atoms were geometrically located using riding models. In θ-CALF-20, the coordinated water

molecule to the Zn^{2+} cation and the pore free molecule were refined with a floating occupancy and the refinement yielded 0.72 and 0.77 as occupancies, respectively. The hydrogens of the free water molecules could not be located satisfactorily from the difference fourier map. CCDC code: 2429210 (θ-CALF-20) contains supplementary crystallographic data for this study. The data can be obtained free of charge from the Cambridge Crystallographic Data Center.

3. Home-built gas loading setup

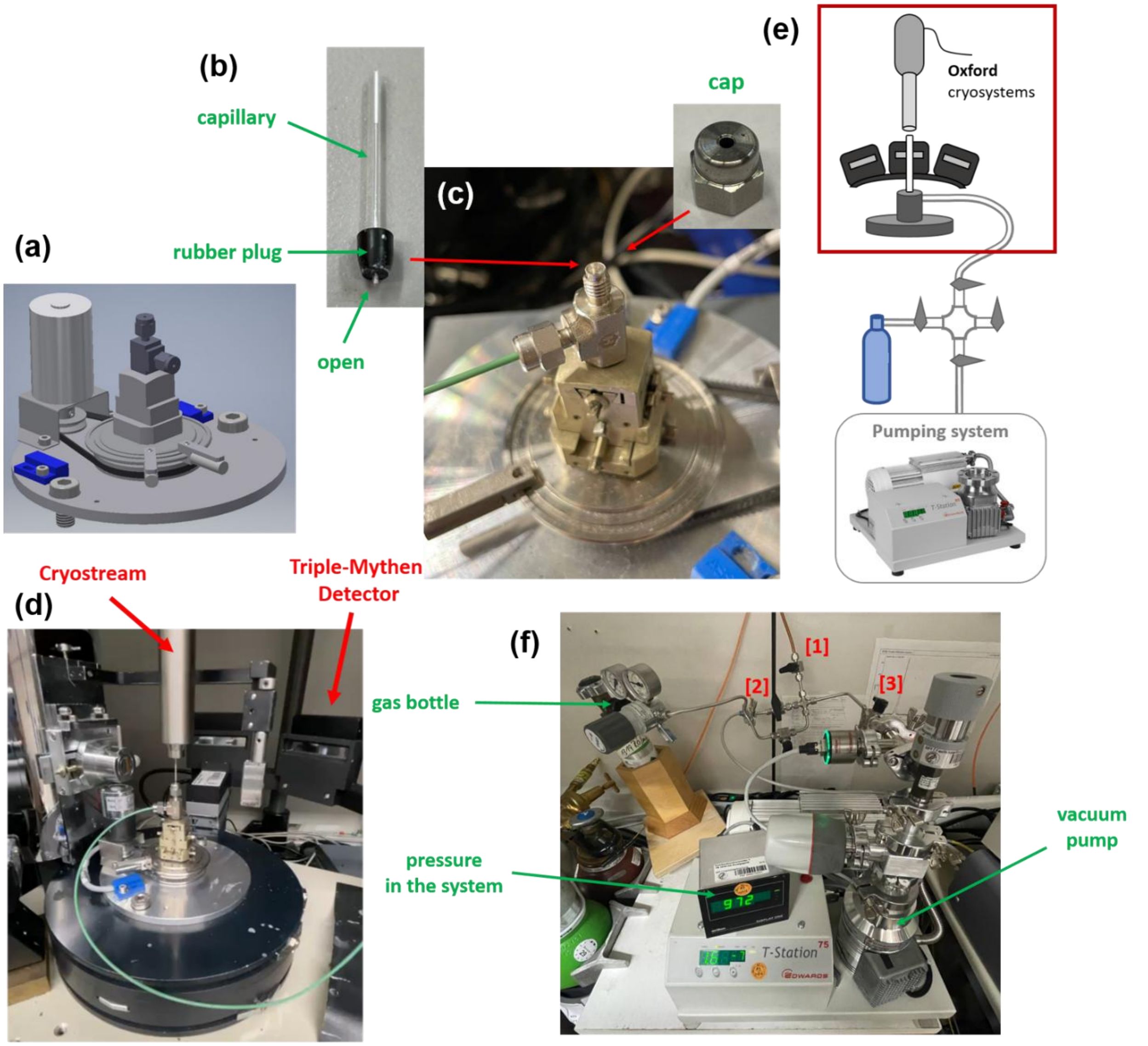

Figure S 1. Home-built gas loading setup (a) schematic 3D model of the sample holder attached to an electro motor which enables alternating rotation, (b) borosilicate glass capillary with rubber plug for sealing, (c) photograph of the sample holder, (d) photograph with the sample holder integrated into a STOE Stadi-P diffractometer with gas tube attached, (e) schematic representation of the gas loading setup, (f) photograph of the pipe and valve system attaching the vacuum pump and the gas supply to the sample holder.

4. Characterization of the CALF-20 MOF

The reflections in the XRPD pattern of as-synthesized CALF-20 (Figure S 2, a, black line) cannot be assigned to the known[1] crystal structure. As the pattern also cannot be indexed, we assume that the as synthesized sample exhibits a multiphase character due to different solvent uptake stages. Heating under dynamic vacuum ($p < 1 \cdot 10^{-2}$ mbar) leads to a drastic change in the diffraction pattern (Figure S 2, a, red line), which can be now indexed by the primitive, monoclinic unit cell of CALF-20 published by Lin *et al*.[1]. Upon cooling, the XRPD pattern does not show further changes (blue line). Due to the remarkable crystallinity of CALF-20, the pattern can be subjected to a high quality Rietveld refinement using the published crystal structre[1] as starting model. The resulting fit (Figure S 2, b) shows a good match between calculated and measured patterns and satisfying agreement factors (R-wp = 3.49 %, GOF. = 2.88). As there are no unindexed nor poorly described peaks and as no residual electron density could be found in the pores of CALF-20, we conclude, that activation led to a pure phase with (within the detection limit) empty pores.

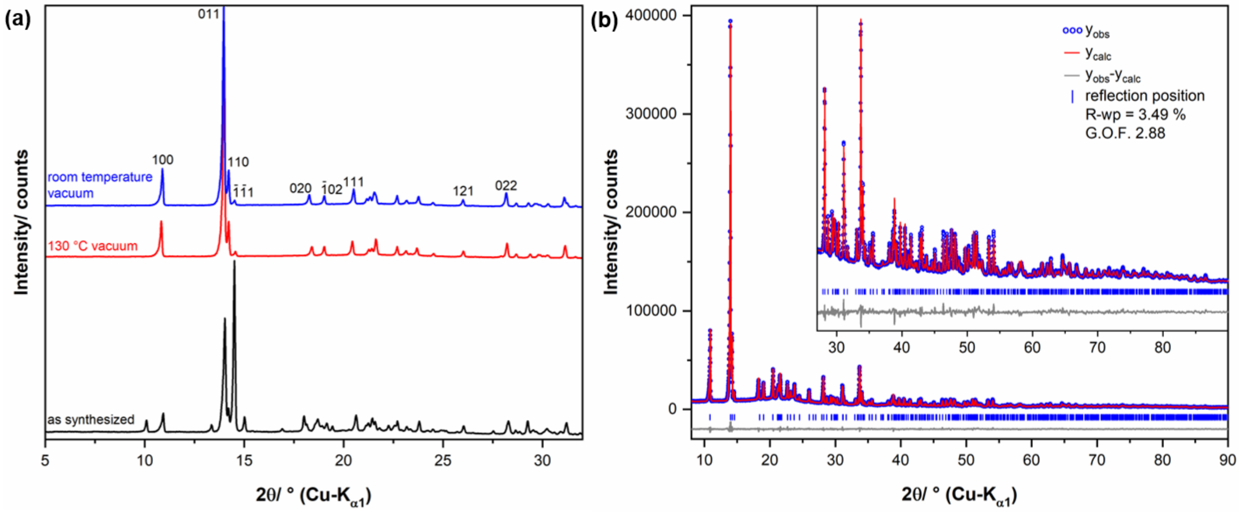

Figure S 2. (a) Excerpts from XRPD patterns of CALF-20 recorded as synthesized (black line), during the activation by heating under dynamic vacuum at 130 °C (red line) and after activation at room temperature under vacuum (blue line), (b) graphical result of the final Rietveld refinement of activated CALF-20, in the inset, the 2θ range above 27 ° is enlarged by a factor of 9 for clarity.

5. Thermal expansion of CALF-20 under vacuum

In order to disentangle the lattice deformation of CALF-20 induced by host molecules from the thermal expansion, we investigated the thermal expansion under dynamic vacuum in detail. While heating, a clear peak shift can be observed, which indicates a considerable thermal expansion (Figure S 3, a), which is highly anisotropic as indicated by the downshift of the 001 and the upshift of the 020 reflection. Between -70 °C and 130 °C, evacuated and activated CALF-20 shows four thermal expansion regimes (Figure S 3, b, I-IV). Below -40 °C, in [100] and [001] direction a positive thermal expansion occurs, whereas negative thermal expansion predominates in *b*-direction, which leads to an overall negative volume expansion of -

29(3) K^{-6} (Figure S 3, b, c, Table S 1). Between -40 °C and -10 °C, the expansion in *a*-direction continues homogenously, whereas the *b*-axis also shows positive thermal expansion and the negative thermal expansion perpendicular to the *ab*-plane tremendously increases, which in the end leads to an increase in the negative volume expansion coefficient by a factor of 2.5 (Figure S 3, b, d, Table S 1). At around 0 °C, the thermal expansion behaviour changes drastically: whereas the *a*- and *b*- axes contract, the 001 lattice plane distances largely expands, which leads to a total positive volume expansion. Moreover, the strong lattice deformation leads to a reduction in the space group symmetry to $P\overline{1}$ (Figure S 3, b, open symbols). The refined α and γ angles of the triclinic cell are very close to 90 ° and the overall cell metric is very similar to the monoclinic counterparts (Table S 2), any attempt to fit the XRPD pattern using a monoclinic cell failed (Figure S 4). As we only obtained two data points during the expansion regime III, we abstained from calculating the linear thermal expansion coefficients. Above 10 °C, the thermal expansion regime is comparable to regime I, with a positive thermal expansion occurring in [100]- and [001]-direction, whereas the *b*-axes shows a negative thermal expansion, which leads to a total negative volume expansion with comparable expansion coefficients (Figure S 3, b, e, Table S 1). We found that the kink in the thermal expansion behaviour that materializes in expansion regimes II and III is quite reproducible during cooling. Heating of the evacuated MOF leads to varying results with the observed discontinuity in thermal expansion behaviour occurring at different temperatures in the range between -20 °C and 20 °C.

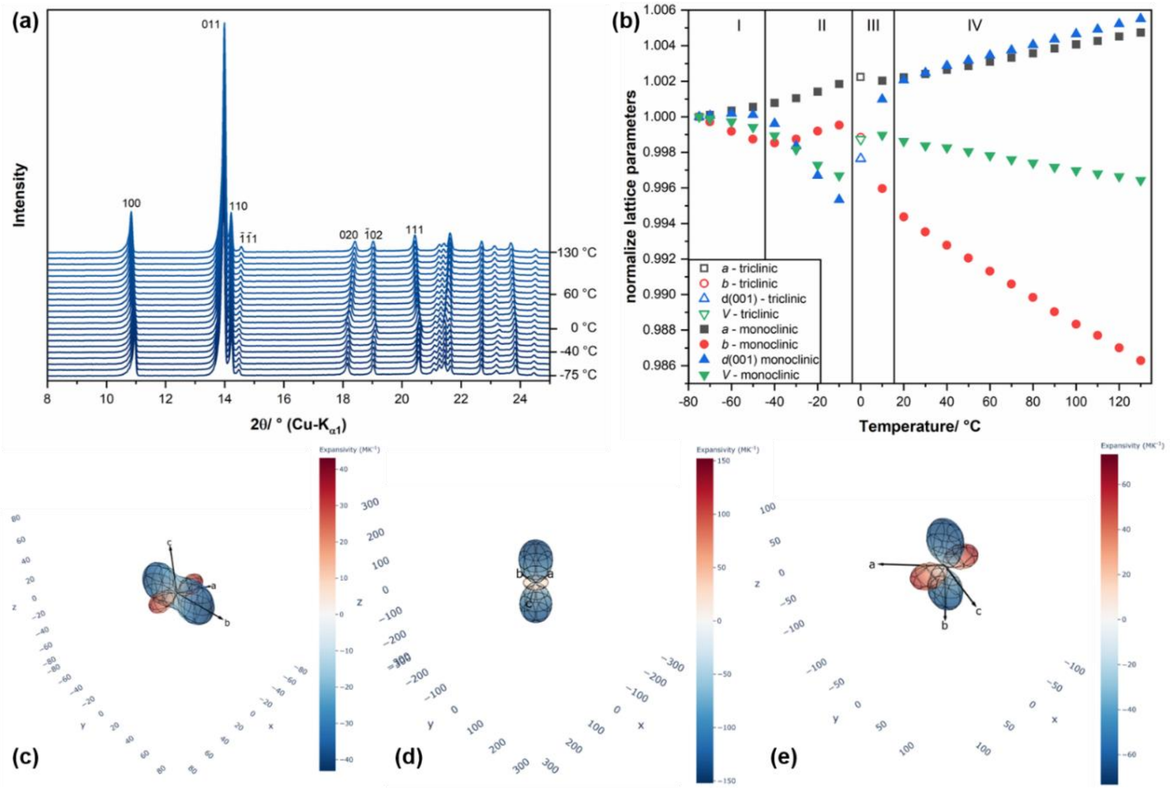

Figure S 3. (a) Excerpts from temperature dependent *in situ* XRPD patterns of activated CALF-20 recorded while cooling under dynamic vacuum (p < 1 · 10^{-2} mbar) including selected reflection indices, (b) evolution of the normalized lattice parameters obtained from fully weighted Rietveld refinements of activated CALF-20 while cooling under dynamic vacuum (p < 1 · 10^{-2} mbar), closed symbols indicate primitive, monoclinic cells, open symbols indicate primitive, triclinic cells, different thermal expansion regimes are indicated by Roman numbers from I to IV; plots showing the variation of the thermal expansion coefficient α with the direction during thermal expansion Phase I (c), Phase II (d) and Phase (IV) created by using the PASCAL software[2], where red lines indicate positive and blue lines negative thermal expansion.

Table S 1. Volume expansion coefficients and all axes expansion coefficients of activated CALF-20 during cooling under dynamic vacuum (p < 1 · 10^{-2} mbar) during thermal expansion Phase I, II and IV calculated using the PASCAL software[2], α is the linear coefficient of the thermal expansion with σ(α) being the corresponding estimated standard deviation, a, b and c are the projections the principal directions X_n on the unit cell axes.

**axes**	**α/ K^{-6}**	***a***	***b***	***c***
		**Phase I (-75 °C - -50 °C)**		
**X1**	33(2)	0.9567	0.0000	0.2910
**X2**	-43(4)	0.0000	1.0000	0.0000
**X3**	-19(5)	0.1640	0.0000	0.9865
**V**	-29(3)			
		**Phase II (-40 °C - -10 °C)**		
**X1**	40(1)	0.9992	0.0000	0.0394
**X2**	39(2)	0.0000	1.0000	0.0000
**X3**	-152(4)	0.3967	0.0000	0.9180
**V**	-73(4)			

Phase IV (20 °C - 130 °C)				
**X1**	59(1)	0.7913	0.0000	0.6115
**X2**	-6(1)	-0.4868	0.0000	0.8735
**X3**	-73(1)	0.0000	1.0000	0.0000
**V**	-20(1)			

Table S 2. Space group and lattice parameters of activated CALF-20 while cooling under dynamic vacuum.

**Temperature/ °C**	**-10**	**0**	**10**
**Space Group**	$P2_1/c(14)$	$P\bar{1}(2)$	$P2_1/c(14)$
***a*/ Å**	9.0598(2)	9.0633(2)	9.0614(2)
***b*/ Å**	9.7514(2)	9.7447(2)	9.7166(2)
***c*/ Å**	9.3002(2)	9.3141(2)	9.3331(2)
***α*/ °**	90	90.082(4)	90
***β*/ °**	116.982(4)	116.889(4)	116.741(4)
***γ*/ °**	90	89.980(4)	90
***V*/ Å³**	732.19(1)	733.68(2)	733.86(2)

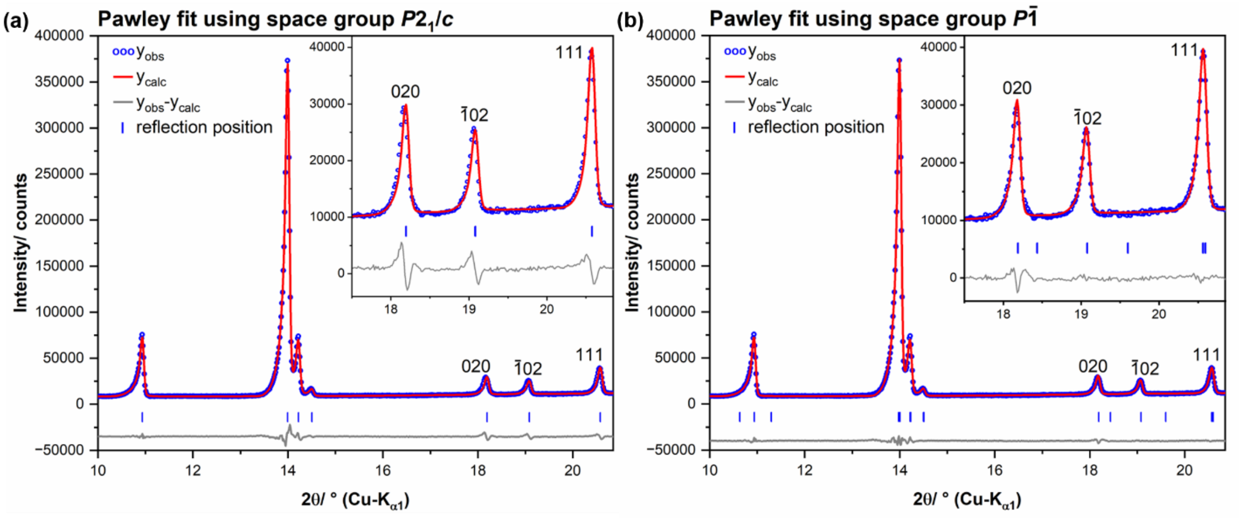

Figure S 4. Graphical results of Pawley refinements of XRPD patterns of activated and evacuated CALF-20 under dynamic vacuum ($p < 1 \cdot 10^{-2}$ mbar) at 0 °C using a (a) monoclinic and (b) a triclinic (**Table S 2**) unit cell.

Additional Figures on CO_2 and H_2O sorption behaviour

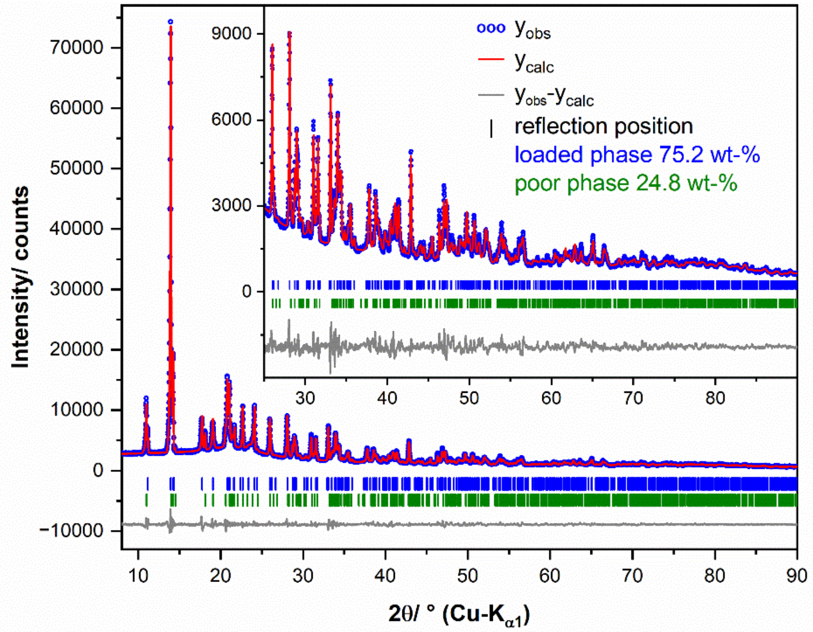

Figure S 5. Graphical result of the final Rietveld refinement (R-wp = 4.98 %) of CALF-20 during isothermal CO_2-loading at -70 °C and p(CO_2) = 142 mbar using two distinct CALF-20 phases: loaded phase with 1.06 mol CO_2 per Zn atom and a = 8.978 Å, b = 9.978 Å, c = 9.306 Å, β = 117.9 °, V = 736.5 Å³ and the loaded phase with 0.04 mol CO_2 per Zn atom and a = 9.027 Å, b = 9.767 Å, c = 9.321 Å, β = 117.0, V = 732.3 Å³. In the inset, the 2θ range above 25 ° is enlarged by a factor of 9 for clarity.

Table S 3. Crystallographic data of CALF-20 while cooling in a pure CO_2 atmosphere (970 mbar).

T in °C	CO_2 loading/ mol per Zn atom	a/ Å	b/ Å	c/ Å	β/ °	d(001)/ Å	V/ Å³
-75	1.10	8.977	9.962	9.340	117.9	8.257	738.4
-70	1.09	8.977	9.961	9.343	117.8	8.262	738.8
-60	1.09	8.976	9.959	9.347	117.8	8.271	739.3
-50	1.09	8.974	9.956	9.354	117.7	8.280	739.9
-40	1.08	8.973	9.955	9.359	117.7	8.290	740.4
-30	1.06	8.970	9.953	9.364	117.6	8.299	740.9
-20	1.04	8.969	9.954	9.365	117.6	8.302	741.2
-10	1.00	8.968	9.956	9.359	117.5	8.299	740.9
0	0.95	8.968	9.959	9.347	117.5	8.289	740.3
10	0.87	8.970	9.963	9.329	117.5	8.273	739.3
20	0.80	8.972	9.966	9.309	117.6	8.253	737.9
30	0.71	8.976	9.967	9.287	117.6	8.232	736.4
40	0.65	8.979	9.966	9.269	117.6	8.214	735.0
50	0.58	8.983	9.963	9.256	117.6	8.201	733.9
60	0.51	8.986	9.955	9.248	117.6	8.195	733.2
70	0.47	8.991	9.943	9.248	117.6	8.198	732.9
80	0.45	9.000	9.924	9.256	117.5	8.212	733.5
90	0.38	9.007	9.898	9.268	117.4	8.231	733.8
100	0.33	9.013	9.8670	9.279	117.2	8.250	733.9

T in °C	CO_2 loading/ mol per Zn atom	a/ Å	b/ Å	c/ Å	β/ °	d(001)/ Å	V/ Å³
110	0.30	9.021	9.845	9.289	117.1	8.267	734.2
120	0.28	9.030	9.814	9.299	117.0	8.286	734.3
130	0.21	9.039	9.785	9.309	116.9	8.303	734.4

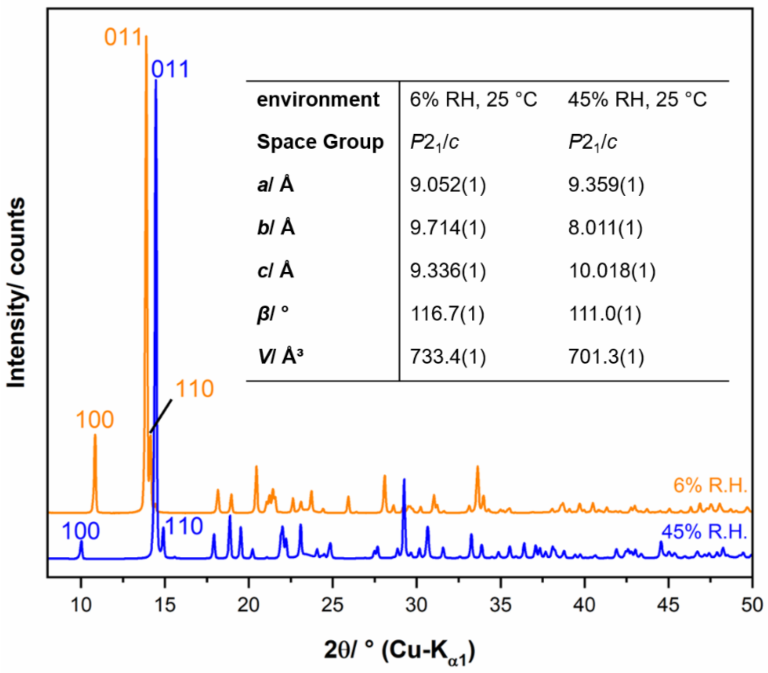

Figure S 6. Comparison of the measured *in situ* XRPD patterns of CALF-20 during the exposure to a dynamic nitrogen atmosphere with 6 % R.H. (orange) and 45 % R.H. (blue) including selected reflection indices and the space group and refined lattice parameters (inset).

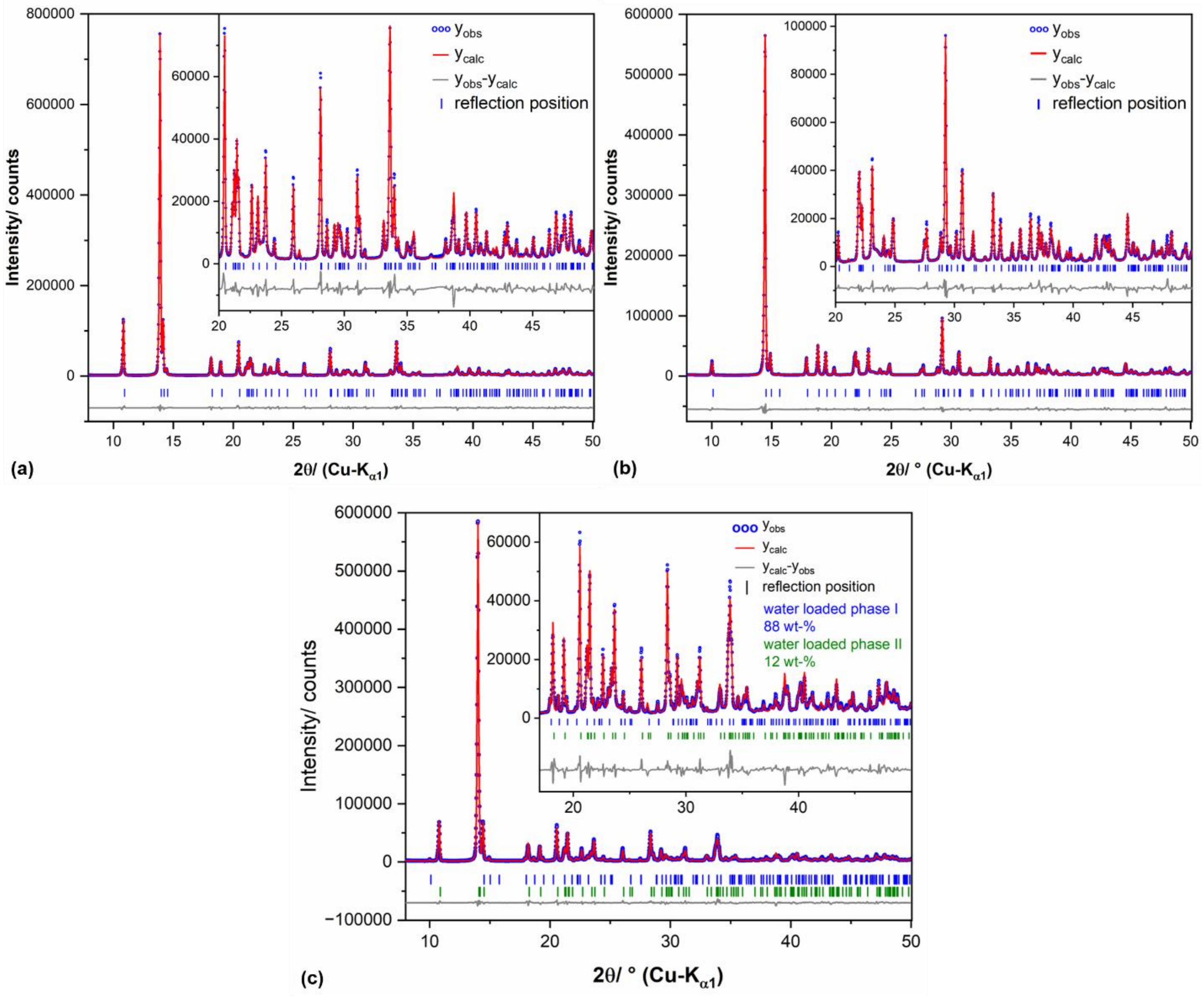

Figure S 7. Graphical result of the final Rietveld refinements of the crystal structures of CALF-20 during the exposition to (a) 6 % R.H., R-wp = 4.56 % and (b) 46 % R.H., R-wp = 5.19 %. and (c) 25 % R.H., R-wp = 5.43 % at 25 °C. In the insets, the 2θ starting at 20 ° is enlarged for clarity.

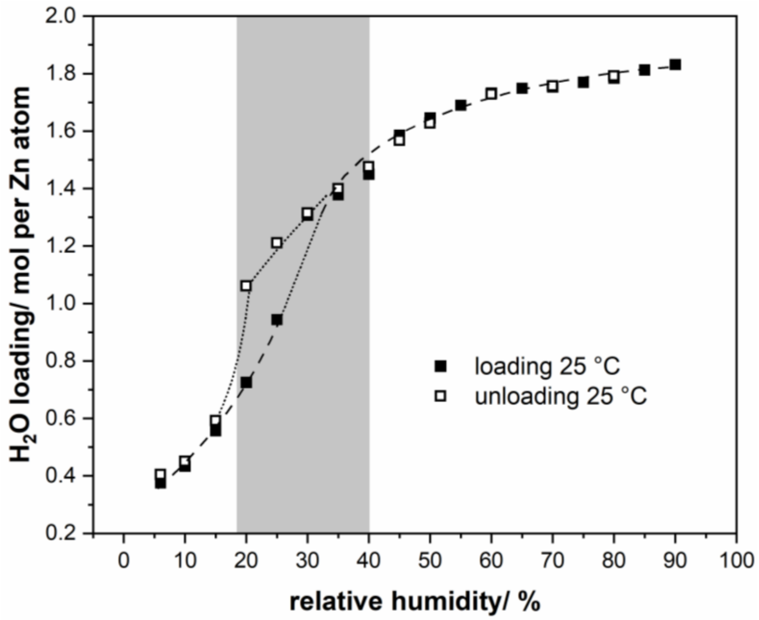

Figure S 8. Evolution of the bulk water content of CALF-20 during increasing (filled symbol) and decreasing (open symbols) the relative humidity of the dynamic atmosphere. The grey shaded area highlights the existence of two distinct, crystalline, hydrated CALF-20 phases I and II (Main Text, Figure 4, a, II).

6. Crystallographic details

Exposing α-CALF-20 crystals to ambient condition induced a SC-to-SC phase transition, resulting in the formation of θ-CALF-20 (Figure S 9 a).

Interestingly, the simulated XRPD patterns generated from θ-CALF-20 and τ-CALF-20 crystallographic information files align perfectly with that of previously published β-CALF-20 (Figure S 9 b).[3] Despite similar simulated XRPD patterns, there are disparities between Zn^{2+} coordination environment in θ- and τ-CALF-20 (six-fold coordination) compared to that of β-CALF-20 (four-fold coordination with a *bis*-monodentate oxalate). Table S3 compares the unit cell parameters of a variety of CALF-20 polymorphs (derived either from powder or single crystal X-ray Diffraction studies) with θ-CALF-20 and H_2O loaded Phase II CALF-20 in the current study. Table S 4. reports on crystallographic data and structure refinement for θ-CALF-20.

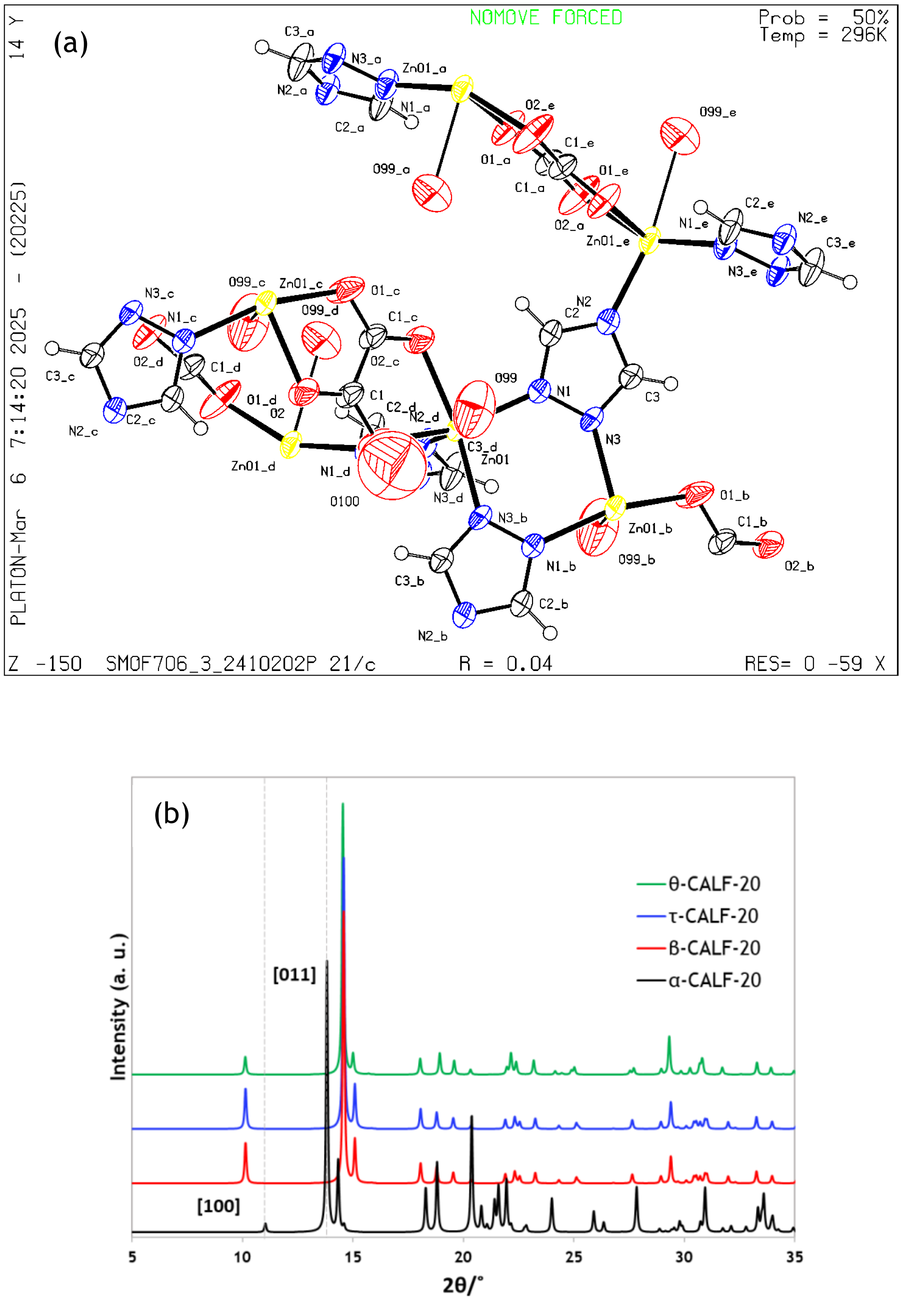

Figure S 9. (a) θ-CALF-20 crystal structure obtained by SC-XRD with a free and a Zn-coordinated H_2O. Color code: Zn- Yellow, C- Grey, O - Red, N - Blue, H- White. b) XRPD pattern comparison of θ-CALF-20 with previously published α-CALF-20[1], β-CALF-20[3] and τ-CALF-20[4] . β-CALF-20 was obtained from XRPD studies.

Table S 4. Unit cell parameters for θ-CALF-20 and comparison with hydrated phases.

**MOF**	**α-CALF-20**[1]	**θ-CALF-20**	**H_2O loaded CALF-20 Phase II**	**τ-CALF-20**[4]	**β-CALF-20**[4]
**Space group**	$P2_1/c$	$P2_1/c$	$P2_1/c$	$P2_1/c$	$P2_1/c$
**a/ Å**	8.9138(12)	9.3356(2)	9.359(1)	9.3052(4)	9.278838
**b/ Å**	9.6935(12)	8.0085(2)	8.011(1)	7.9498(3)	7.933906
**c/ Å**	9.4836(13)	10.0154(3)	10.018(1)	10.0430(5)	10.039276
**β/ °**	115.895(4)	110.661(2)	111.0(1)	110.117(5)	109.89147
**V/ Å³**	737.17(17)	700.634	701.27(1)	697.60 (6)	694.972

Table S 5. Crystallographic data and structure refinement for ***θ-CALF-20***.

**Empirical formula**	$Zn_2(C_2O_4)(C_2N_3H_2)_2(H_2O)_{1.5}.(H_2O)_{1.5}$
**Formula weight**	200.96
**Temperature/K**	296 K
**Crystal system**	Monoclinic
**Space group**	$P2_1/c$
***a*/Å**	9.3356(2) Å
***b*/Å**	8.0085(2) Å
***c*/Å**	10.0154(3) Å
***β*/°**	110.661(2)°
**Volume/Å³**	700.634 Å³
Z	4
**ρ_{calc} /g cm³**	1.905
**μ/mm⁻¹**	4.644
**Radiation**	Cu K-α
***θ* range for data collection/°**	5.063 to 68.23
**Index ranges**	-11 =< h =< 11, -9 =< k =< 9, -12 =< l =< 12
**Reflections collected**	10695
**Independent reflections**	1286
**Goodness-of-fit on F²**	1.253
**Final *R* indexes [*I*>=2σ (I)]**	R1 = 0.0418, wR2 = 0.0942
**Final *R* indexes [all data]**	R1 = 0.0656, wR2 = 0.0970
**Largest diff. peak/hole / e Å⁻³**	-0.58

7. Computational Method

To understand the interactions between the CALF-20 structure and both coordinated water and free water, we employed a multi-step simulation approach involving density functional theory (DFT)[5] calculations and molecular dynamics. DFT calculations were performed using the FHI-aims[6] program with the Perdew-Burke-Ernzerhof[7] (PBE) generalized gradient approximation (GGA) for exchange-correlation effects. A tight numerical atom-centered basis set ("tier 2") was used for both MOF elements and the guest molecule to ensure high accuracy. Noncovalent (Van der Waals) interactions were accounted for using the exchange-dipole model (XDM) post-SCF dispersion correction [A. D. Becke and E. R. Johnson (2005) J. Chem. Phys. 122, pp. 154104, E. R. Johnson and A. D. Becke (2005) J. Chem. Phys. 123, pp. 024101]. The Brillouin zone was sampled with a 5×5×5 k-point mesh, ensuring force convergence within 1 meV/Å.

Additionally, molecular dynamics simulations were conducted using path-integral molecular dynamics (PIMD) [R. P. Feynman, A. R. Hibbs, Quantum Mechanics and Path Integrals (Dover Publications, Mineola, NY, 2005),[8] to capture nuclear quantum effects. Guest molecule (water) binding sites within the pore were identified through an in-house semi-automated search, systematically exploring at least 20 locations. The system was then equilibrated at 295 K in an NVT ensemble. Binding energy was determined by calculating the energies of the isolated guest molecule, the relaxed MOF framework, and the equilibrated composite system, using the equation:

$$E_b = E_{MOF+guest} - \left(E_{MOF} + E_{guest}\right). \quad \text{(Eq. 1)}$$

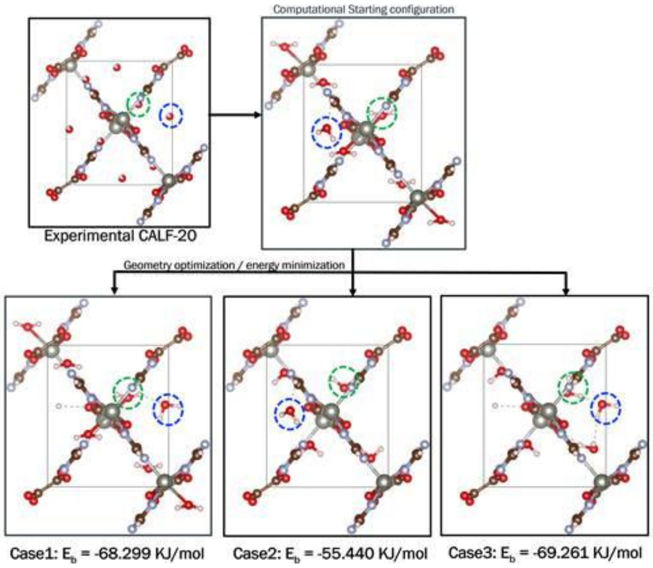

Figure S 10. Crystal structures (showing the atomic positions) in the experimental CALF-20 solved using the single crystal x-ray diffractometer (SCXRD) and the binding energy in different water coordination cases. The blue dashed circle represents the free water position, while the green dashed circle indicates the coordinated water position.

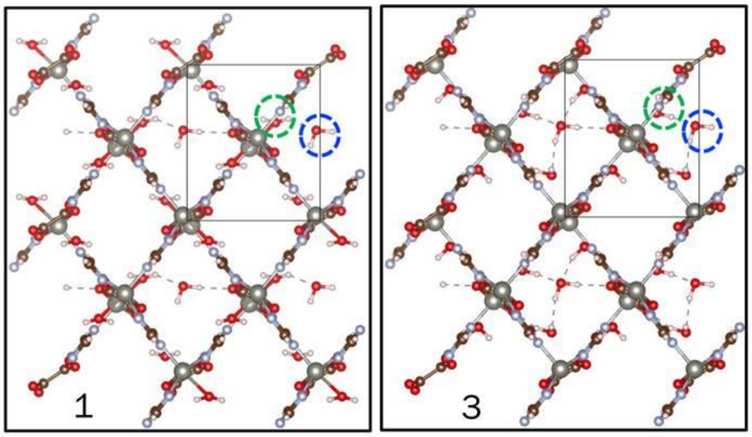

Figure S 11. A 2x2x2 supercell for case 1 and 2 illustrating the formation of water-water and water-oxalate chains through hydrogen bonding.

CALF20 Isotherms- CO_2 & Water

Method description:

CO_2 adsorption isotherms of CALF-20 were measured using a standard volumetric method over a temperature range of 273–387 K with a 3Flex instrument (Micromeritics Instruments Inc.). Prior to gas adsorption, all samples (~100 mg) were degassed under vacuum at 140°C for 5 hours to remove any adsorbed species. The isotherm at 273 K was measured in physisorption mode, whereas chemisorption mode was used for higher-temperature measurements. The full dataset is presented in Figure S12, where

(a) shows the CO_2 isotherm at 273 K with low-pressure data highlighted in the inset on a logarithmic scale, and (b) displays higher temperature isotherms at 323 K, 343 K, 363 K, and 387 K.

(a)

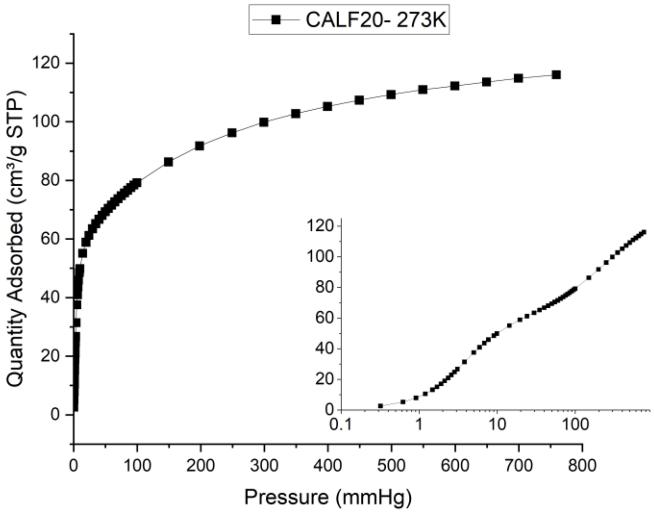

(b)

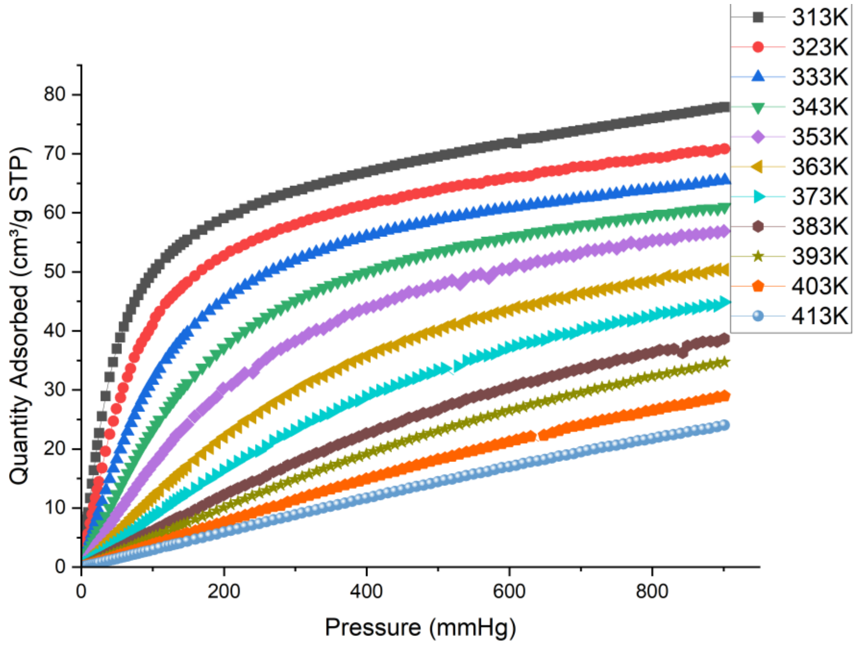

Figure S 12. (a) CO_2 isotherm at 273K. inset shows low pressure data using logarithmic scale x-axis, (b) Higher temperature CO_2 isotherms (323, 343, 363 and 387 K)

Method description:

The water isotherm of CALF-20 was measured using a gravimetric vapor sorption analyzer (DVS Resolution, Surface Measurement Systems). Samples were activated under a dry nitrogen flow at 140 °C for 2 hours before measurement. A Dm/dt value of 0.0005%/min was used as the equilibrium criterion for each data point, ensuring accurate sorption measurements.

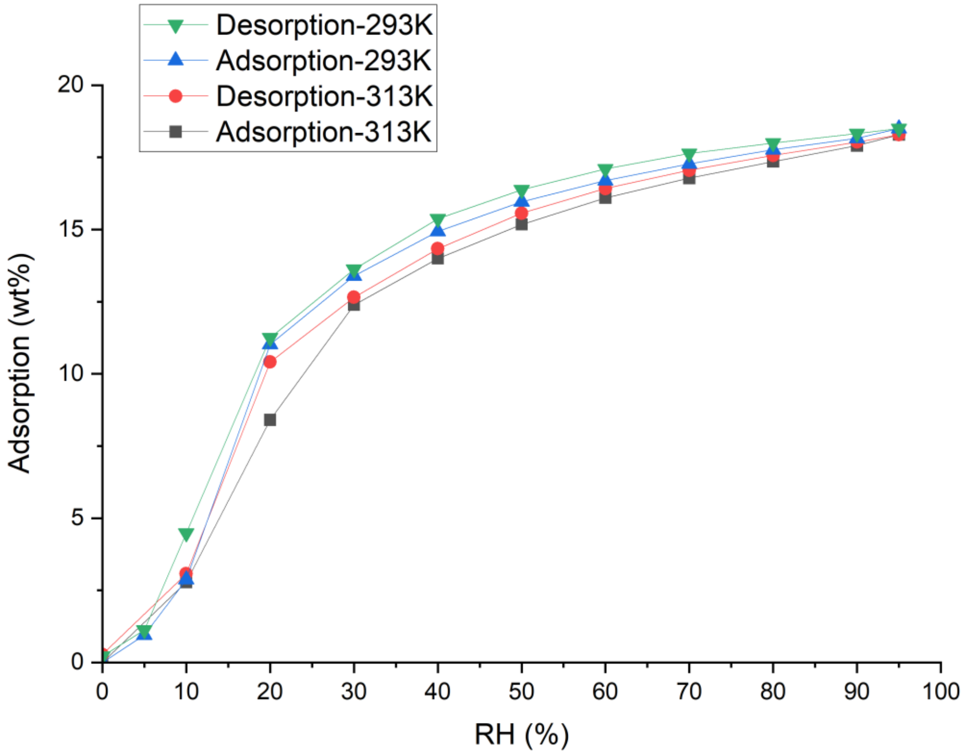

Figure S 13. H_2O isotherm at two temperatures (293 and 313K).

Table S 6. Comparison of the crystal structure data of reported CALF-20 forms. This table is an addition an extension of Table S1 in the supporting information of the study by Drwęska et al.[4]. Forms exhibiting vast similarities in terms of unit cell metrics and loading state are highlighted by red, green and orange background color, respectively.

**Phase**	**α-CALF-20**	**α-CALF-20-act**	**CALF-20-act-evac**[c]	**α-CALF-20-CO_2**	**CO_2-loaded CALF-20**[c]	**β-CALF-20**	**γ-CALF-20**	**τ-CALF-20**	**θ-CALF-20**	**H_2O loaded CALF-20 Phase II**[c]	**H_2O loaded CALF-20 Phase I**[c]
**Reference**	Drwęska [4]	Drwęska [4]	This work	Drwęska [4]	This work	Chen[3]	Drwęska [4]	Drwęska [4]	This work	This work	This work
**T/ K**	296(2)	296(2)	303(2)	296(2)	293(2)	173(2)	140.1(2)	296(2)	296(2)	298(1)	298(1)
**P/mbar**	≈ 970?*	?*	$< 1\cdot10^{-3}$	10^4 ?*	≈ 970	≈ 970?*	≈ 970?*	≈ 970?*	≈ 972	≈ 972	≈ 972
**sample environment**	?*	static/ dynamic vacuum?*	dynamic vacuum	static CO_2 atmosphere	static CO_2 atmosphere	dynamic atmosphere?*	crystal oil	crystal oil	crystal oil	dynamic atmosphere, 40 % R.H.	dynamic atmosphere, 20 % R.H.
**Loading/ mol per Zn atom**	N.A.	No loading	No loading	0.5 mol CO_2	0.8 mol CO_2	x mol H_2O	0.5 mol H_2O	0.5 mol H_2O	1.5 mol H_2O	1.5 mol H_2O	0.7 mol H_2O
**Method**	SC-XRD	SC-XRD	XRPD	SC-XRD	XRPD	PXRD	SC-XRD	SC-XRD	SC-XRD	XRPD	XRPD
**Space group**	$P2_1/c$	$P2_1/c$	$P2_1/c$	$P2_1/c$	$P2_1/c$	$P2_1/c$	$P2_1/c$	$P2_1/c$	$P2_1/c$	$P2_1/c$	$P2_1/c$
***a*/ Å**	8.9375(2)	8.9765(2)	9.0649(2)	8.9367(3)	8.9726(1)	9.2788(4)	9.3919(5)	9.3052(4)	9.3356(2)	9.3446(2)	9.0728(2)
***b*/ Å**	9.7321(2)	9.7142(3)	9.6928(2)	9.8435(3)	9.9673(2)	7.9340(3)	7.5264(4)	7.9498(3)	8.0085(2)	7.9928(2)	9.7528(3)
***c*/ Å**	9.5429(2)	9.4546(3)	9.3380(2)	9.4623(3)	9.3038(2)	10.0393(4)	10.2078(6)	10.0430(5)	10.0154(3)	10.0288(3)	9.2646(3)
***β*/ °**	115.679(1)	116.217(1)	116.63(1)	116.636(1)	117.574(1)	109.893(5)	110.078(6)	110.117(5)	110.661(2)	110.717(2)	117.071(2)
**V/ Å³**	748.07(3)	739.62(4)	733.43(2)	744.04(4)	737.56(2)	695.0(5)	677.71(7)	697.60(6)	700.63(4)	700.61(3)	729.96(3)(
**Zn-coordi-nation**[a]	2xO, 3xN	2xO, 3xN	2xO, 3xN	2xO, 3xN	2xO, 3xN	2xO, 3xN	2xO, 3xN, 1xOw	2xO, 3xN, 1xOw	2xO, 3xN, 1xOw	2xO, 3xN, 1xOw	2xO, 3xN/ 2xO, 3xN, 1xOw[b]

*information cannot be extracted unambiguously from the literature

[a]we included all possible up to a distance of 2.5 Å into the coordination sphere of zinc

[b]if for high water loading (achieved by high temperatures and high relative humidity, main text, Figure 4d), an additional water mole enters the coordination sphere of zinc in H2O loaded CALF-20 Phase I

[c]CIF files of these CALF-20 structure are included into the ESI, for Rietveld plots see Figure S 14-Figure S 17

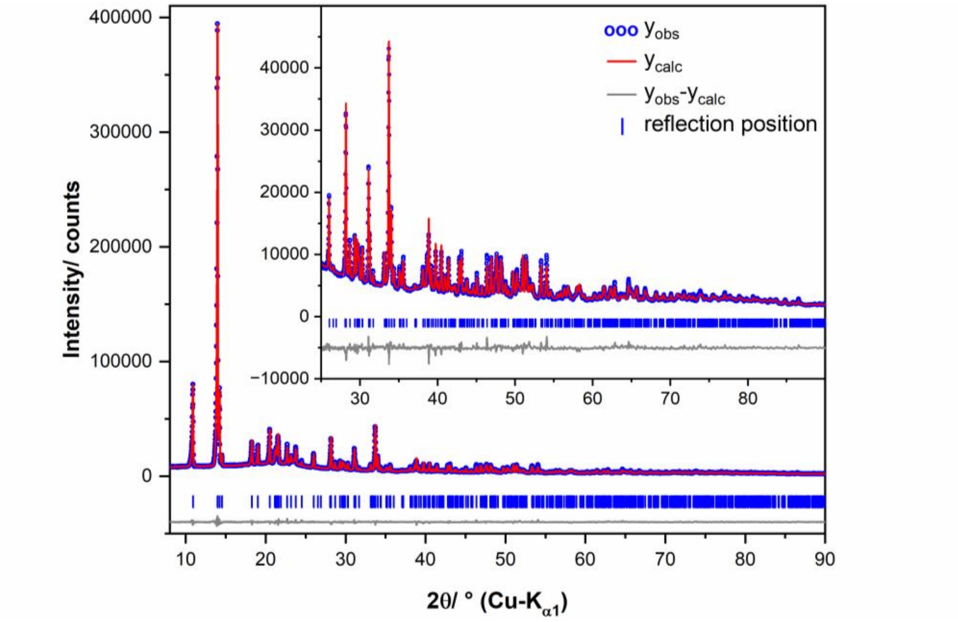

Figure S 14. Graphical result of the final Rietveld refinement of the crystal structures of CALF-20 measured under dynamic vacuum (p = < $1 \cdot 10^{-3}$ mbar) at 30 °C (= CALF-20-act-evac, Table S 6) after the activation under dynamic vacuum at 130 °C, R-wp = 3.24 %.

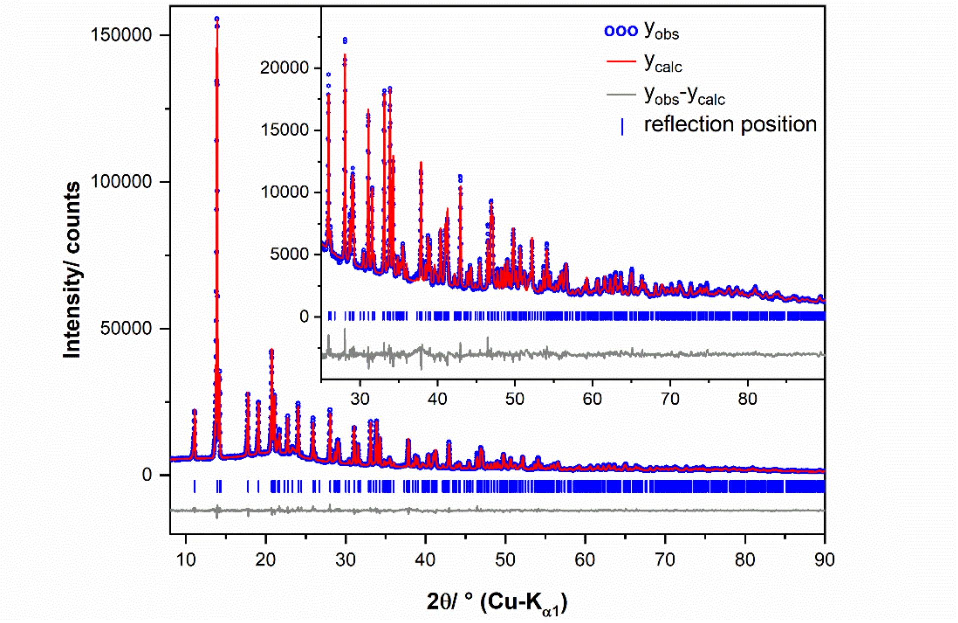

Figure S 15. Graphical result of the final Rietveld refinement of the crystal structures of CALF-20 measured under static CO2 atmosphere (p ≈ 970 mbar) at 20 °C (= CO_2-loaded CALF-20, Table S 6), R-wp = 3.55 %.

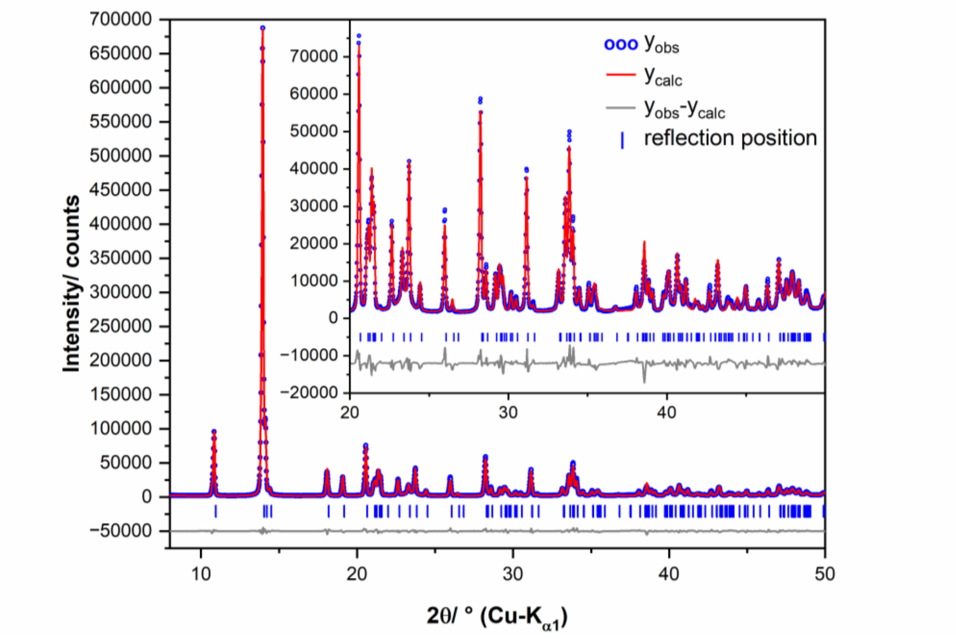

Figure S 16. Graphical result of the final Rietveld refinement of the crystal structures of CALF-20 measured under dynamic nitrogen atmosphere with 20 % R.H. at 25 °C (= H_2O loaded CALF-20 Phase I, Table S 6), R-wp = 4.57 %.

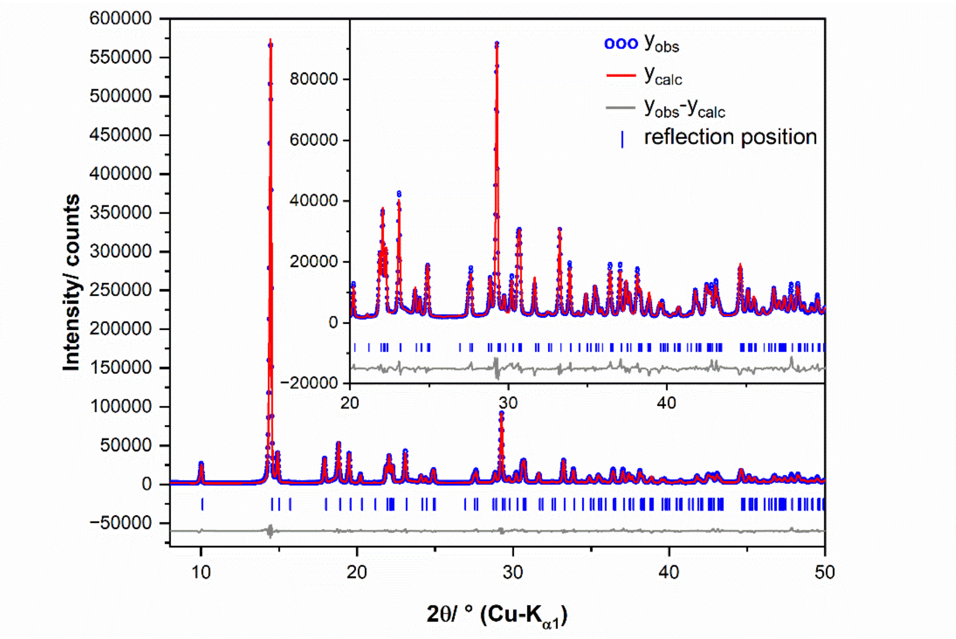

Figure S 17. Graphical result of the final Rietveld refinement of the crystal structures of CALF-20 measured under dynamic nitrogen atmosphere with 40 % R.H. at 25 °C (= H_2O loaded CALF-20 Phase II, Table S 6), R-wp = 4.57 %.

References

(1) Lin, J.-B.; Nguyen, T. T. T.; Vaidhyanathan, R.; Burner, J.; Taylor, J. M.; Durekova, H.; Akhtar, F.; Mah, R. K.; Ghaffari-Nik, O.; Marx, S.; et al. A scalable metal-organic framework as a durable physisorbent for carbon dioxide capture. *Science* **2021**, *374* (6574), 1464-1469. DOI: 10.1126/science.abi7281.
(2) Cliffe, M. J.; Goodwin, A. L. PASCal: a principal axis strain calculator for thermal expansion and compressibility determination. *Journal of Applied Crystallography* **2012**, *45* (6), 1321-1329. DOI: 10.1107/s0021889812043026.
(3) Chen, Z.; Ho, C.-H.; Wang, X.; Vornholt, S. M.; Rayder, T. M.; Islamoglu, T.; Farha, O. K.; Paesani, F.; Chapman, K. W. Humidity-Responsive Polymorphism in CALF-20: A Resilient MOF Physisorbent for CO2 Capture. *ACS Materials Letters* **2023**, *5* (11), 2942-2947. DOI: 10.1021/acsmaterialslett.3c00930.
(4) Drwęska, J.; Formalik, F.; Roztocki, K.; Snurr, R. Q.; Barbour, L. J.; Janiak, A. M. Unveiling Temperature-Induced Structural Phase Transformations and CO2 Binding Sites in CALF-20. *Inorganic Chemistry* **2024**, *63* (41), 19277-19286. DOI: 10.1021/acs.inorgchem.4c02952.
(5) Kohn, W.; Sham, L. J. Self-Consistent Equations Including Exchange and Correlation Effects. *Physical Review* **1965**, *140* (4A), A1133-A1138. DOI: 10.1103/PhysRev.140.A1133.
(6) Blum, V.; Gehrke, R.; Hanke, F.; Havu, P.; Havu, V.; Ren, X.; Reuter, K.; Scheffler, M. Ab initio molecular simulations with numeric atom-centered orbitals. *Computer Physics Communications* **2009**, *180* (11), 2175-2196. DOI: 10.1016/j.cpc.2009.06.022.
(7) Perdew, J. P.; Burke, K.; Ernzerhof, M. Generalized Gradient Approximation Made Simple. *Physical Review Letters* **1996**, *77* (18), 3865-3868. DOI: 10.1103/PhysRevLett.77.3865.
(8) Parrinello, M.; Rahman, A. Study of an F center in molten KCl. *The Journal of Chemical Physics* **1984**, *80* (2), 860-867. DOI: 10.1063/1.446740. Hirshberg, B.; Rizzi, V.; Parrinello, M. Path integral molecular dynamics for bosons. *Proceedings of the National Academy of Sciences* **2019**, *116* (43), 21445-21449. DOI: 10.1073/pnas.1913365116.
